# H9N2 virus-derived M1 protein promotes H5N6 virus release in mammalian cells: Mechanism of avian influenza virus inter-species infection in humans

**DOI:** 10.1371/journal.ppat.1010098

**Published:** 2021-12-03

**Authors:** Fangtao Li, Jiyu Liu, Jizhe Yang, Haoran Sun, Zhimin Jiang, Chenxi Wang, Xin Zhang, Yinghui Yu, Chuankuo Zhao, Juan Pu, Yipeng Sun, Kin-Chow Chang, Jinhua Liu, Honglei Sun

**Affiliations:** 1 Key Laboratory of Animal Epidemiology, Ministry of Agriculture, College of Veterinary Medicine, China Agricultural University, Beijing, China; 2 School of Veterinary Medicine and Science, University of Nottingham, Sutton Bonington Campus, United Kingdom; University of Georgia, UNITED STATES

## Abstract

H5N6 highly pathogenic avian influenza virus (HPAIV) clade 2.3.4.4 not only exhibits unprecedented intercontinental spread in poultry, but can also cause serious infection in humans, posing a public health threat. Phylogenetic analyses show that 40% (8/20) of H5N6 viruses that infected humans carried H9N2 virus-derived internal genes. However, the precise contribution of H9N2 virus-derived internal genes to H5N6 virus infection in humans is unclear. Here, we report on the functional contribution of the H9N2 virus-derived matrix protein 1 (M1) to enhanced H5N6 virus replication capacity in mammalian cells. Unlike H5N1 virus-derived M1 protein, H9N2 virus-derived M1 protein showed high binding affinity for H5N6 hemagglutinin (HA) protein and increased viral progeny particle release in different mammalian cell lines. Human host factor, G protein subunit beta 1 (GNB1), exhibited strong binding to H9N2 virus-derived M1 protein to facilitate M1 transport to budding sites at the cell membrane. *GNB1* knockdown inhibited the interaction between H9N2 virus-derived M1 and HA protein, and reduced influenza virus-like particles (VLPs) release. Our findings indicate that H9N2 virus-derived M1 protein promotes avian H5N6 influenza virus release from mammalian, in particular human cells, which could be a major viral factor for H5N6 virus cross-species infection.

## Author summary

H9N2 avian influenza viruses (AIVs), through reassortment of their internal genes with other circulating AIVs subtypes (H5N6, H7N9, H10N8 and H10N3 viruses), are known to enable the resulting novel reassortants to infect humans. However, the precise roles of H9N2 virus-derived internal genes in human infection remain unknown. Here, we found that H9N2 virus-derived (but not H5N1 virus-derived) M1 protein showed strong interaction with host GNB1 protein to enhance M1 protein transport to budding sites at the cell membrane to promote progeny virus release, hence facilitating H5N6 reassortants to infect human cells.

## Introduction

AIVs pose a constant threat to public health by regularly crossing host-species barriers to infect humans. H5N1 HPAIV has caused at least 863 reported human cases at around 53% mortality[1]. Since the first H5N6 virus infection in humans in China in 2014, there are to date 47 reported infections and 24 deaths[2]. H7N9 AIVs emerged in China in 2013 and subsequently caused five influenza epidemics[3]. Avian H9N2 viruses circulate globally through wild birds and are endemic in domestic poultry in China and elsewhere. Recent studies indicate that H9N2 viruses are capable of binding to the human-type sialic acid (SA) receptor and are transmissible among ferrets via respiratory droplets[4]. As of December 2020, at least 66 global cases of H9N2 infections in humans have been reported[5].

Genetic drift and genetic reassortment are two types of evolutionary changes that occur in influenza viruses[6]. The G57 genotype of H9N2 AIVs is regarded as a principal donor of viral genes through reassortment with co-circulating influenza viruses and resulted in zoonotic reassortants (H5N6, H7N9, H10N8 and H10N3 viruses)[7–12], indicating that the H9N2 virus-derived internal genes are crucial in the cross-species transmission of AIVs to humans. Among such reassortants, H7N9, H10N8 and H10N3 viruses are endemic in China; while H5N6 viruses have been reported in several countries carried by migratory birds[13–15]. Globally, from 2015 to 2021, there were only 7 reported cases of human infection with avian influenza A (H5N1) virus, while 45 new human cases of avian influenza A (H5N6) virus infection were reported to the World Health Organization (WHO), suggesting that the H5N6 virus poses a bigger threat to public health[2]. In addition, H5N6 viruses have also been isolated from other mammals, including cats and pigs[16,17]. Furthermore, clade 2.3.4.4 H5N6 viruses can bind both avian- and human-like SA receptors and have higher transmissibility than H5N1 viruses in experimental ferrets[18–21]. All these findings indicate that H5N6 viruses pose a serious threat to public health. Therefore, whether the H9N2 virus-derived internal genes confer a replication advantage to H5N6 viruses in mammalian hosts needs to be determined.

A key feature of the G57 H9N2 virus is the replacement of the earlier A/chicken/Beijing/1/1994 (BJ/94)-like *M* gene with the A/quail/Hong Kong/G1/1997 (G1)-like *M* gene of quail origin[22]. The *M* gene regulates multiple processes during influenza A virus replication through the M1 protein and the proton channel protein (M2)[23,24]. The M1 protein is the most abundant viral protein responsible for the structural shell of the virus linking the viral envelope with the nucleocapsid[23]; it is involved in the shuttling of the viral ribonucleoprotein (vRNP) complex between the nucleus and cytoplasm during viral replication[25–27]. When bound to HA and neuraminidase (NA), M1 protein serves as a docking site for vRNP and M2 recruitment to virus budding sites, which polymerize and form the emerging virion. Hence, M1 protein provides structure to the virion and bridges interactions between the viral lipid membrane and the vRNP core for virus assembly and budding[28,29]. The M2 protein is an integral membrane protein in the viral envelope with proton channel activity[24]. M1 and M2 proteins are thus essential in influenza virus assembly and budding, and determine virus morphology[23]. Mutations in *M* gene have been found to be critical in affecting viral replication, pathogenicity and cross-species infection[30–32].

Here, we report on cross-species transmission role of the H9N2 virus-derived M1 protein in conferring H5N6 viruses with higher replication efficiency in mammalian cells. Human host factor, GNB1, showed enhanced binding to H9N2 virus-derived M1 protein to promote M1 transport to the cell membrane which, in turn, facilitated the interaction between M1 and HA proteins, ultimately improving viral assembly and release.

## Results

### Reassortment of H9N2 virus-derived *M* gene enhanced H5N6 virus replication in mammalian cells

**Fig 1:**
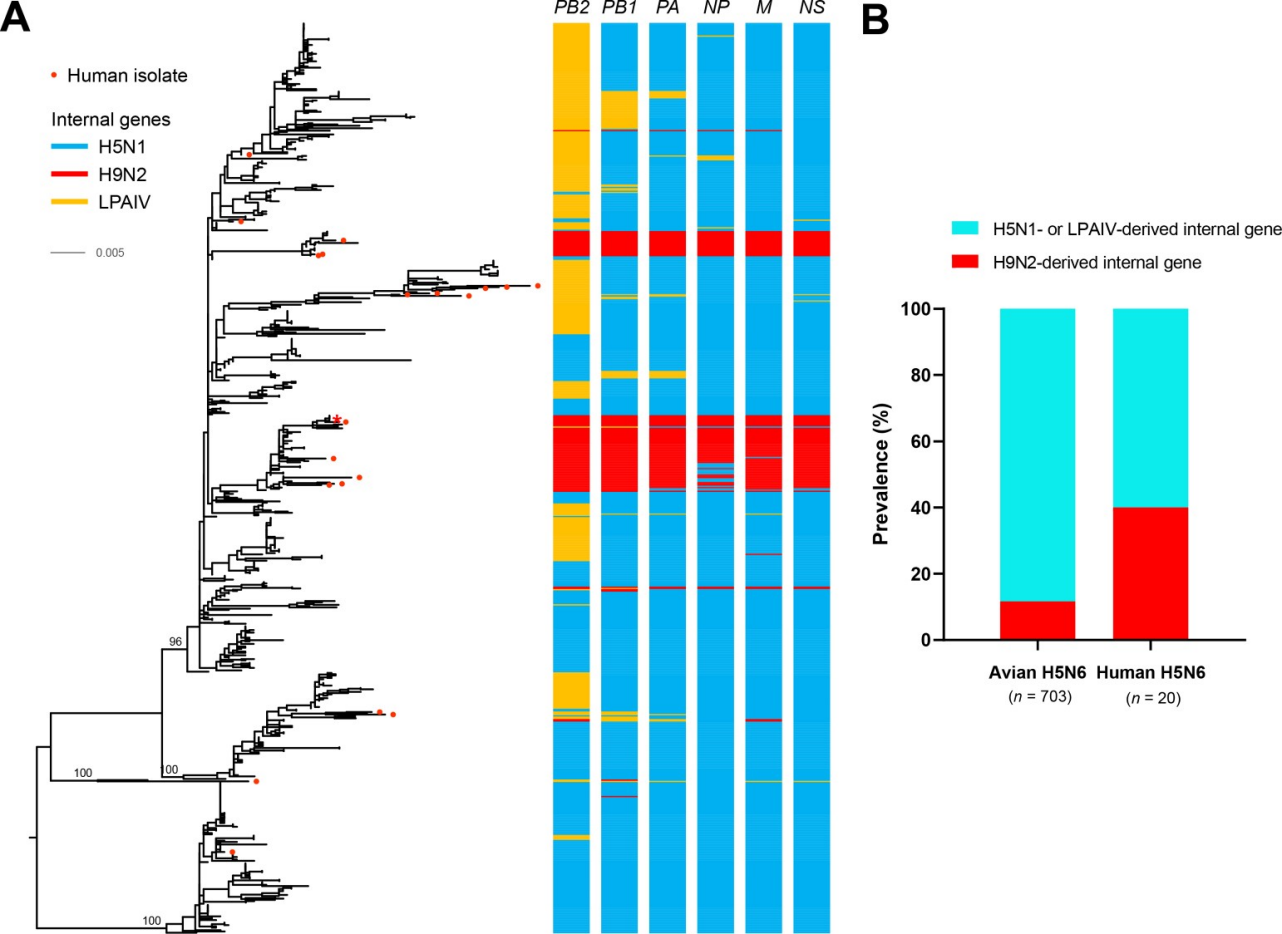
Phylogenetic analysis of H5N6 viruses. (A) Phylogenetic analysis of H5N6 viruses in China. The clade origins of each internal gene are indicated by different colored bars. Detailed phylogenetic trees are available as S1 Fig and S1 Table. H5N6 human strains are marked as red circles, virus labeled with a red asterisk was used in the present research. (B) Frequencies of H5N6 viruses carrying H9N2 virus-derived internal genes of human and avian origin.

Presently, the number of reported human cases of H5N6 virus infection is 47, and the genome sequences of 20 cases have been ascertained. Phylogenetic analysis of H5N6 viruses, using RAxML[33], found that the internal genes could be grouped into multiple genotypes based on the genetic reassortment with H5N1 virus, H9N2 virus, or waterfowl-origin low pathogenic avian influenza virus (LPAIV) (Figs 1A and S1 and S1 Table). Further analysis showed that 11.7% (82/703) of H5N6 viruses circulating in poultry carried reassorted H9N2 virus-derived internal genes, while H5N6 viruses that infected humans carried reassorted H9N2 virus-derived internal genes at 40.0% (8/20) (Fig 1B).

**Fig 2:**
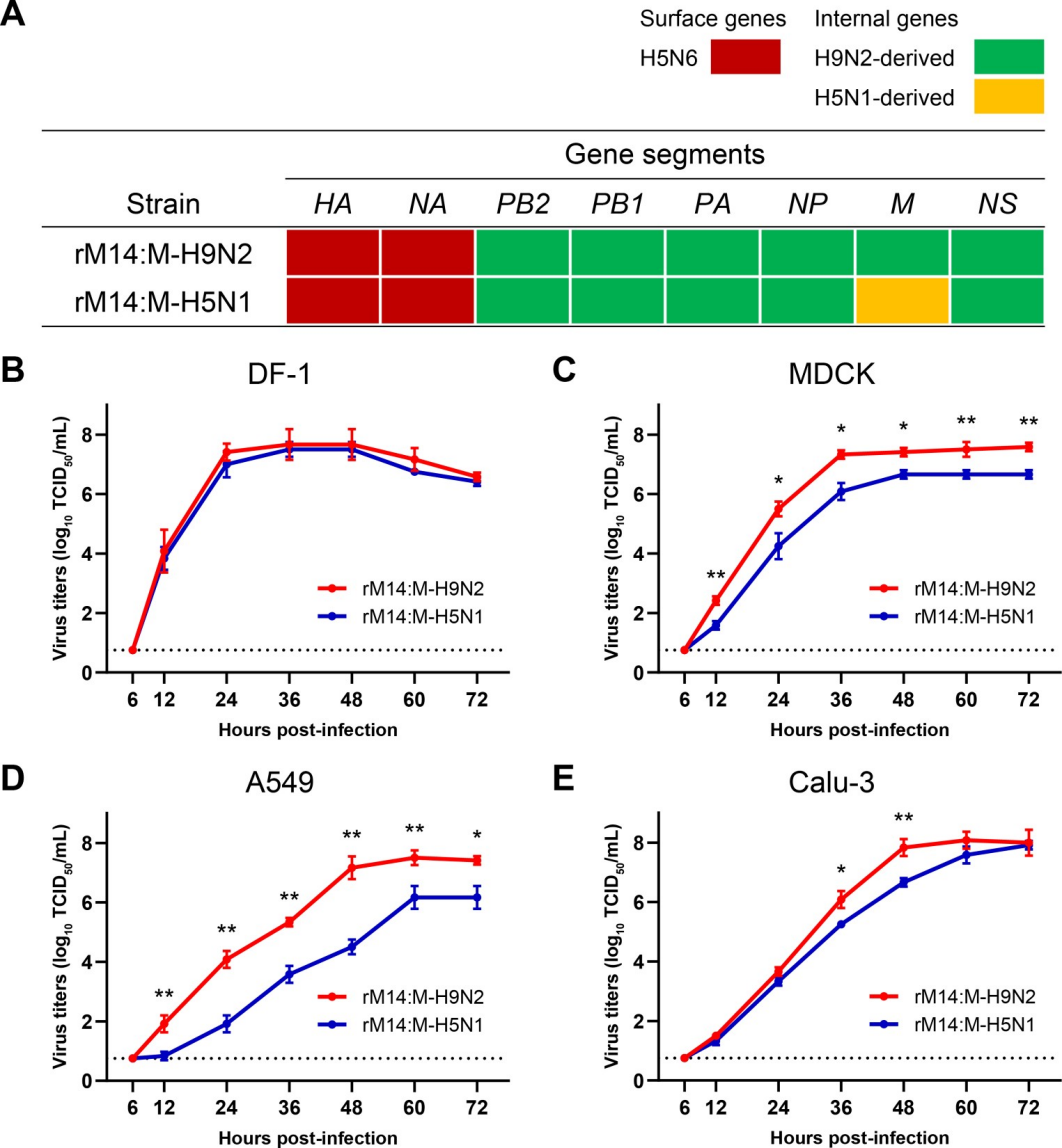
Reassortment of the H9N2 virus-derived *M* gene enhanced H5N6 virus replication in mammalian cells. (A) Gene origins of rM14:M-H9N2 and rM14:M-H5N1 viruses. (B-E) Multistep growth curves of rM14:M-H9N2 and rM14:M-H5N1 viruses in DF-1, MDCK, A549 and Calu-3 cells, respectively (MOI of 0.01). Virus titers were determined from the supernatants collected at the indicated time points. Statistical significance was based on one-way ANOVA (*, *P* < 0.05; **, *P* < 0.01).

To investigate the contribution of the H9N2 virus-derived *M* gene to H5N6 virus replication in mammalian cells, a reverse genetics-derived H5N6 virus (rM14:M-H9N2), from a wild-type H5N6 isolate (A/goose/Northern China/M14/2016) containing H9N2 virus-derived internal genes, was generated. Eight genes of the M14 virus shared high nucleotide identity with human-isolated H5N6 viruses possessing H9N2-derived internal genes, with identities ranging from 96.3 ± 2.2% (for the *PB2* gene) to 98.6 ± 0.6% (for the *M* gene), indicating a consistent genotype (S2 Table). Another virus reassortant with H5N1-derived *M* gene (rM14:M-H5N1) in the rM14 virus background was also generated (Fig 2A). Multistep replication kinetics assays of rM14:M-H9N2 and rM14:M-H5N1 were performed with a multiplicity of infection (MOI) of 0.01 in embryonic chicken fibroblast cells (DF-1), Madin-Darby canine kidney cells (MDCK), human airway epithelial cells (Calu-3), and human alveolar basal epithelial cells (A549) over 72 h. There was no difference in virus titers between the two viruses in DF-1 cells (Fig 2B). However, MDCK and A549 cells infected with rM14:M-H9N2 virus produced more infectious virus than those infected with rM14:M-H5N1 virus from 12 h post-infection (hpi) (*P* < 0.05). At their peak, the mean output of rM14:M-H9N2 virus were up to 10-fold higher than that of rM14:M-H5N1 virus (Fig 2C and 2D). In Calu-3 cells, at 36 to 48 hpi, rM14:M-H9N2 virus produced more viable progeny viruses (*P* < 0.05) than those of rM14:M-H5N1 virus (Fig 2E). Together, these results suggest that the H9N2 virus-derived *M* gene improves the ability of H5N6 virus to replicate in mammalian cells.

### Enhanced binding interaction between H9N2 virus-derived M1 and H5N6 HA proteins in mammalian cells

**Fig 3:**
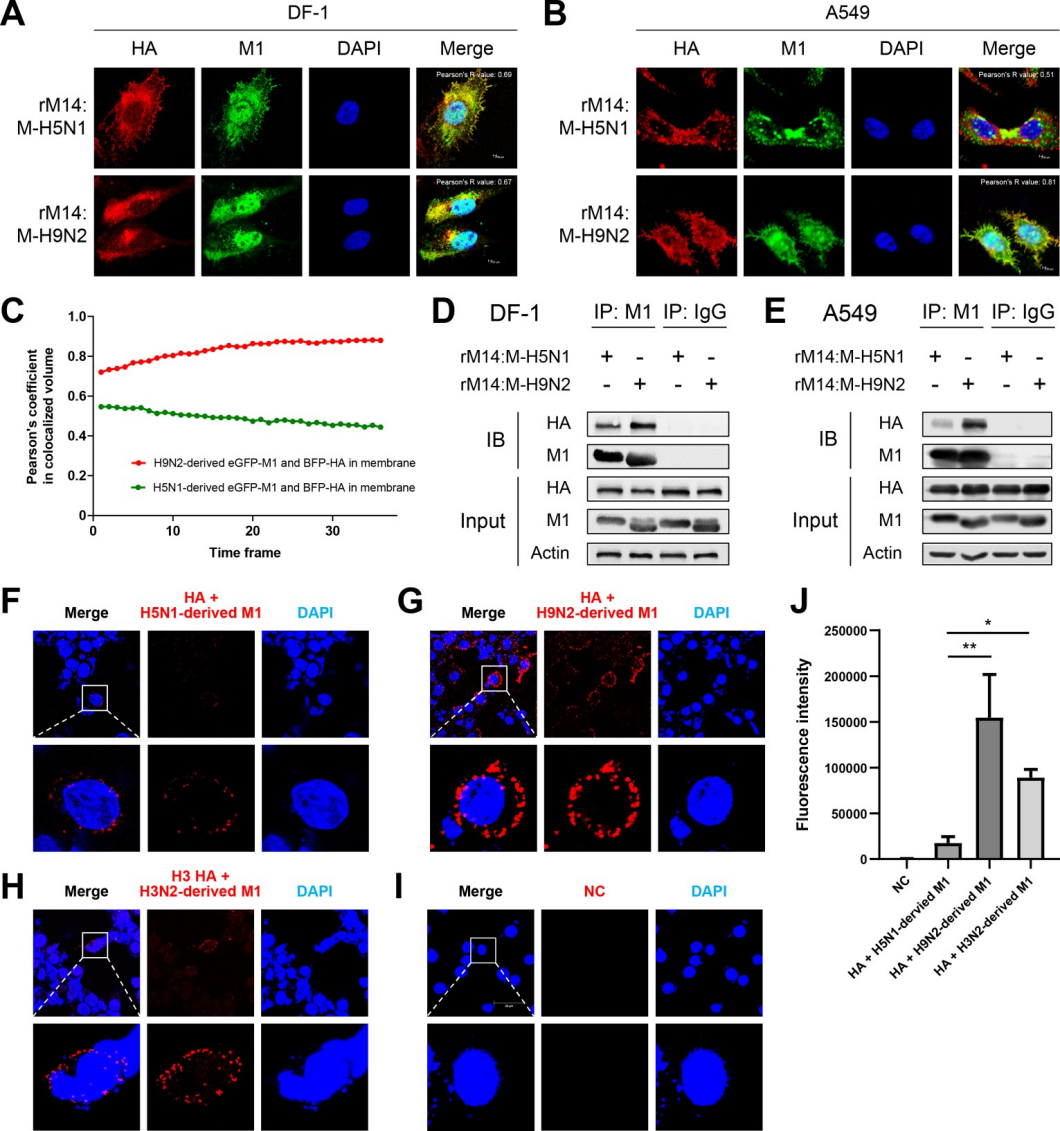
H9N2 virus-derived M1 protein strongly bound HA protein in mammalian cells. (A-B) DF-1 or A549 cells were infected with rM14:M-H5N1 or rM14:M-H9N2 viruses at an MOI of 1. Confocal microscopy was performed to detect H5N6 HA and M1 protein localization in cells (green and red fluorescence resulting in a yellow color denote colocalization). (C) The dynamic colocalization 24 h post-transfection as observed via live imaging of H9N2 virus-derived *eGFP-M1/BFP-HA* or H5N1-derived *eGFP-M1/BFP-HA* transfected A549 cells. Pearson’s coefficients were analyzed in co-localized volume between eGFP-M1 and BFP-HA located at the membrane in 36-time frames (1-time frame duration = 42.62 s). (D–E) Physical interaction of viral M1 and HA proteins in cells. (D) DF-1 or (E) A549 cells separately infected with rM14:M-H5N1 and rM14:M-H9N2 viruses at an MOI of 1. At 24 hpi, cell lysates were immunoprecipitated using anti-M1 antibody or anti-IgG antibody, followed by Western blotting for influenza M1 and HA proteins. In A549 cells, an increased binding ability was observed between H9N2-derived M1 and HA proteins than between H5N1-derived M1 and HA proteins. IB, immunoblot. (F–H) interaction between influenza HA and M1 protein was determined by proximity ligation assay. 293T cells were co-transfected with (F) H5 *HA* and H5N1-derived *M1*, (G) H5 *HA* and H9N2-derived *M1*, (H) H3 *HA* and H3N2-derived *M1*, or (I) empty vector as negative control (NC). 24 h post transfection, PLA was performed using antibodies specific to influenza M1 and HA proteins. Fluorescence of cells was analyzed by a fluorescence confocal microscope (red fluorescent signal). Nuclei were stained with DAPI (blue). (J) Multiple images (F–I) were processed by BlobFinder software to measure the PLA fluorescence intensity (~30 cells total for each condition). Graphs show the means ± SD of three independent experiments (*, *P* < 0.05; **, *P* < 0.01).

M1 and HA proteins promote the budding of progeny influenza viruses[34,35]. To investigate whether H5N6 HA showed differences in co-localization with H9N2-derived and H5N1-derived M1, confocal microscopy was performed on DF-1 and A549 cells separately infected with the two recombinant H5N6 viruses (rM14:M-H5N1 and rM14:M-H9N2, MOI = 1). In DF-1 cells, there was no difference in the co-localization of HA and M1 proteins in cells infected with each virus (Fig 3A). However, in A549 cells, rM14:M-H9N2 virus infection exhibited visibly stronger co-localization of HA and M1 proteins than that of rM14:M-H5N1 virus infection (Fig 3B).

To examine the co-localization dynamics between the viral M1 and HA proteins at the plasma membrane of A549 cells, we performed live imaging at different time points over 24 h post-transfection. Plasma membrane was labeled with CellMask Plasma Membrane 647. Pearson’s coefficient of co-localized volume between H9N2 virus-derived eGFP-M1 and BFP-HA at the plasma membrane ranged from 0.72 to 0.88 in 36-time frames, while that between H5N1-derived eGFP-M1 and BFP-HA ranged from 0.55 to 0.44 (Fig 3C). These results indicate that H9N2 virus-derived M1 shows greater co-localization with H5N6 HA at the plasma membrane.

Next, we determined the specific interaction between M1 and HA proteins using co-immunoprecipitation (Co-IP) assay. In DF-1 cells, the binding between H9N2-derived M1 and HA proteins was similar to that of H5N1-derived M1 and HA proteins (Fig 3D). In mammalian cells, greater binding was observed between H9N2-derived M1 and HA proteins than that between H5N1-derived M1 and HA proteins (Figs 3E and S2).

To confirm the physical interaction between M1 and HA proteins in mammalian cells, H5N6 *HA* gene expression plasmids were co-transfected with *M1* gene expression plasmids of different origins (H9N2- or H5N1-derived *M1*) in the human embryonic kidney cell line (293T). Protein interaction was determined by proximity ligation assay (PLA), in which protein interactions were localized using antibodies specific for viral proteins HA and M1. The human seasonal virus A/Kansas/14/2017 (H3N2) was used as control. As shown in the Fig 3F–3J, only faint PLA signals were observed in cells co-transfected with H5N6 *HA* and H5N1-derived *M1* encoding plasmids at 24 h post-transfection (Fig 3F). However, the fluorescence intensity of the PLA signal was significantly higher in the cells co-transfected with H3N2-derived *M1* and H3N2-derived *HA* carrying plasmids (*P* < 0.05) (Fig 3H). In turn, the fluorescence intensity of the PLA signal in the cells co-transfected with *HA* and H9N2-derived *M1* carrying plasmids was the highest compared with the other two viruses (*P* < 0.01) (Fig 3G and 3J).

To identify the key regions where the M1 protein interacts with the HA protein, we truncated the M1 protein to detect interaction of truncated versions with the HA. Results show that the M1 C-terminal domain is a key region for interaction with HA (S3 Fig). Collectively, H9N2 virus-derived M1 shows higher binding affinity than H5N1-derived M1 for H5N6 HA protein in mammalian cells.

### H9N2 virus-derived M1 protein increased viral progeny particle release in mammalian cells

**Fig 4:**
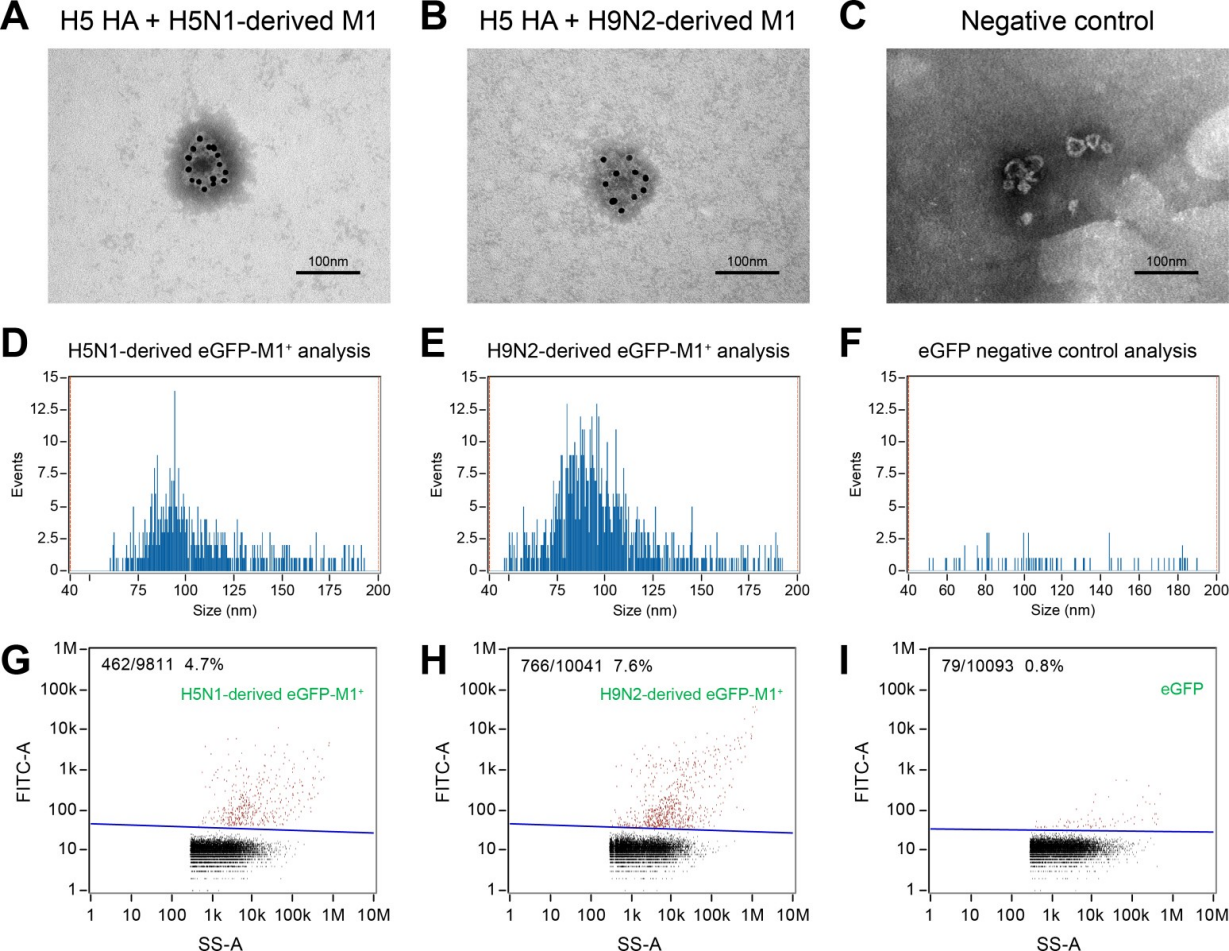
H9N2 virus-derived M1 protein increased viral progeny particle release in mammalian cells. (A–C) IEM analysis of influenza VLPs in the concentrated cell culture supernatant. 293T cells were co-transfected with (A) *HA* and H5N1-derived *M1*, (B) *HA* and H9N2-derived *M1*, or (C) empty vector as negative control. Anti-H5N1 IAV HA antibody was used as the primary antibody. The gold is 10 nm in diameter. (D–I) Size and molecular profiling of eGFP-M1^+^ VLP particles via a Flow NanoAnalyzer. 293T cells were co-transfected with (D, G) *HA* and *H5N1*-derived *eGFP-M1*, (E, H) *HA* and H9N2-derived *eGFP-M1*, or (F, I) empty vector as negative control. eGFP was used to label the VLPs. Size profiling of the purified eGFP-M1^+^ VLP particles with the FITC channel, where Y-axis represents the events, and X-axis represents the size. (G–I) Molecular profiling of eGFP-M1^+^ VLP particles. SS-A, side scatter area.

To assess the effect of interaction between H5 HA and M1 proteins on virus budding and assembly, 293T cells were co-transfected with H5 *HA* gene expression and H9N2-, or H5N1-derived *M1* gene expression plasmids for immune electron microscopy (IEM). H5 *HA* gene transfected-293T cells that co-expressed H9N2 virus-derived *M1* or H5N1-derived *M1* produced VLPs with average diameter of 100 nm, indicating that M1 and HA protein interaction promotes VLP assembly (Fig 4A–4C).

To investigate the role of M1 protein in the assembly and release of influenza viral particles, we performed Nano-Flow cytometry (NanoFCM) to quantify the secreted H5N1-derived eGFP-M1^+^ or H9N2-derived eGFP-M1^+^ VLPs. NanoFCM enables detection of extracellular vesicles (EVs) with a minimum diameter of 40 nm[36–38]. The main peak sizes of eGFP-M1^+^ particles of both groups were approximately 100 nm, similar in size to the diameter of influenza particles observed in IEM (Fig 4D and 4E). The percentage of H5N1-derived eGFP-M1^+^ particles was 4.7% (Fig 4G), whereas that of H9N2-derived eGFP-M1^+^ particles was significantly higher (7.6%, *P* < 0.001) (Fig 4H). Only 0.8% of VLPs from empty vector expressing cells (used as negative control) were eGFP positive (Fig 4F and 4I). These results demonstrate that the interaction of H9N2 virus-derived M1, rather than H5N1 virus-derived M1, with HA protein is more conducive to the release of influenza VLPs.

### Human GNB1 protein exhibited enhanced binding to H9N2 virus-derived M1 protein

Influenza virions contain host proteins that shape virion architecture during budding[39,40]. Liquid chromatography-mass spectrometry (LC-MS) analysis of H9N2 virus-derived M1 complexes identified 154 host proteins that interact with M1 protein (S4A Fig and S3 Table). Using an exponentially modified protein abundance index (emPAI) (≥ 0.4), 28 candidate interacting proteins were shortlisted from the 154 interacting proteins (S4A Fig). Four candidate interacting proteins, karyopherin subunit beta 1 (KPNB1), junction plakoglobin (JUP), cleft lip and palate transmembrane protein 1 (CLPTM1), and GNB1 were further shortlisted with Co-IP assays. These four candidate proteins are connected to the budding process mediated by M1 protein during viral cycle and associated with secretion or transport function[40–44]. Both H9N2- and H5N1-derived M1 proteins specifically bound GNB1; however, H9N2 virus-derived M1 and GNB1 proteins exhibited stronger binding affinity (S4B Fig). However, interaction between H9N2 virus-derived M1 protein with the other three candidate proteins was not evident (S4B Fig).

**Fig 5:**
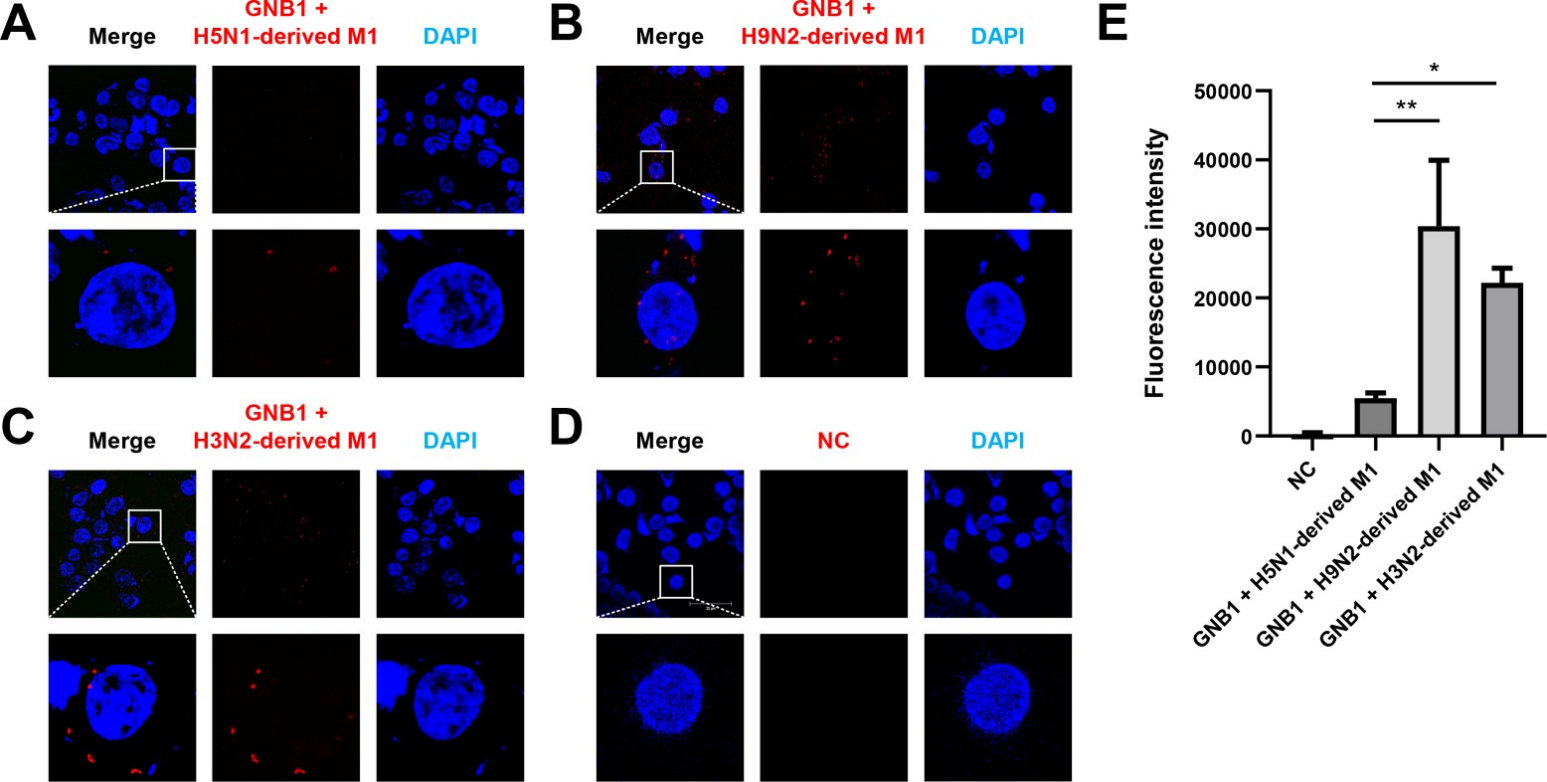
GNB1 protein bound more strongly to H9N2 virus-derived M1 protein than to H5N1-derived M1 protein. (A–D) Interaction between influenza M1 and endogenous GNB1 proteins was determined by PLA. 293T cells were transfected with (A) H5N1-derived *M1* plasmid, (B) H9N2-derived *M1* plasmid, (C) H3N2-derived *M1* plasmid, or (D) empty vector as negative control. The PLA was performed using antibodies specific to influenza M1 and endogenous GNB1 proteins. The fluorescence of cells was analyzed by a fluorescence confocal microscope (red fluorescent signal). Nuclei were stained with DAPI (blue). (E) Multiple images (A–D) were processed by BlobFinder to measure the PLA fluorescence intensity per cell (~30 cells total for each condition). Graphs show means ± SD of three independent experiments (*, *P* < 0.05; **, *P* < 0.01).

To further demonstrate the interaction between H9N2- or H5N1-derived M1 and GNB1 proteins, we performed PLA in 293T cells. Interaction between H3N2-derived M1 and GNB1 proteins was detected as control (Fig 5). Weak PLA signals were found in cells transfected with H5N1-derived *M1* expression plasmid at 24 h post-transfection; in contrast, PLA fluorescence intensity was significantly higher in cells transfected with H3N2-derived *M1* (*P* < 0.05) or H9N2-derived *M1* (*P* < 0.01) (Fig 5). These results indicate that H3N2-derived M1 and H9N2-derived M1 exhibit stronger interaction than H5N1-derived M1 with human GNB1. Therefore, GNB1 is a specific and high affinity protein-binding partner of H9N2 virus-derived M1.

### GNB1 protein facilitated transport of H9N2 virus-derived M1 protein and VLP assembly

**Fig 6:**
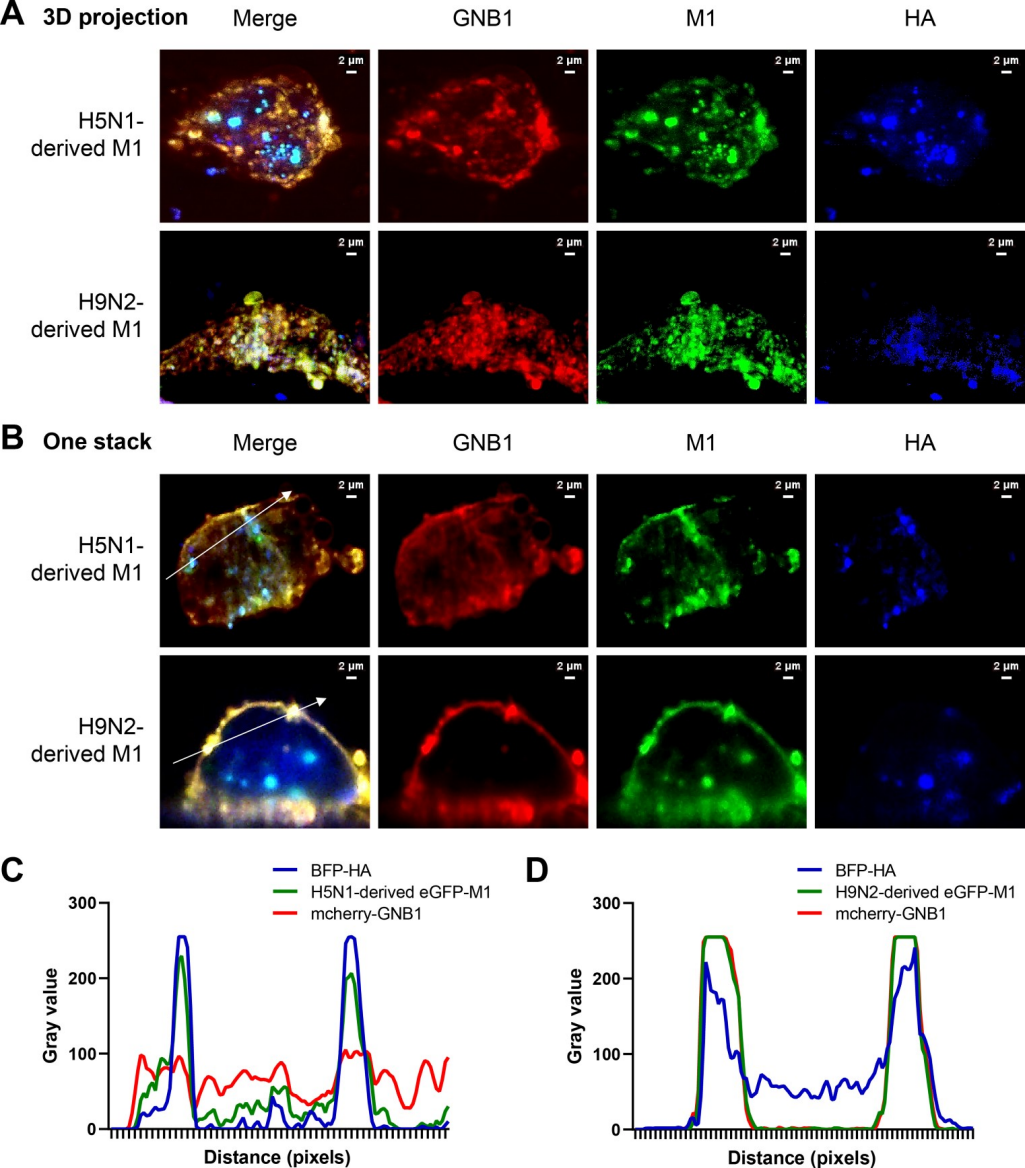
mCherry-GNB1 specifically co-localized with H9N2 virus-derived eGFP-M1 protein. (A–B) Visualization of the single-channel images showing the localization of BFP-HA (blue), eGFP-M1 (green), and mCherry-GNB1 (red) in A549 cells, measured after co-transfecting H9N2-derived *eGFP-M1/BFP-HA/mCherry-GNB1* or H5N1-derived *eGFP-M1/BFP-HA/mCherry-GNB1* in (A) 3D projection or (B) one stack (Z-start: 0.0 μm, Z-end: 50.0 μm, Z-step: 0.5000 μm) using line profiles in image-analysis software platform Fiji. (C–D) The fluorescence intensity profile was plotted along the white arrow crossing the cell membrane in Fig 6B using ImageJ/Fiji software. The signal intensities (absolute gray values from 16-bit raw images) are plotted, where Y-axis shows the signal intensity, and X-axis shows the length of the line. The line profiles from three different channels are merged into one graph. Two different expression patterns are shown, highlighting the differences in the mCherry-GNB1 (red) and the similarities in the BFP-HA (blue) and eGFP-M1 (green) signal. Scale bars: 2 μm.

M1 protein is directed to the apical budding site via its association with HA in lipid rafts[45]. To assess the role of GNB1 and M1 protein interaction in transport capacity of M1 protein, we performed live imaging to observe the dynamics of M1, HA, and GNB1 proteins in A549 cells. The localization of eGFP-M1, BFP-HA, and mCherry-GNB1 in A549 cells was ascertained to determine whether GNB1 protein specifically co-localized with M1 protein in the cell membrane during viral budding. H9N2 virus-derived eGFP-M1, but not H5N1-derived eGFP-M1, extensively co-localized with mCherry-GNB1 (combined green and red fluorescence = yellow color) (Fig 6A). In one stack, the mCherry-GNB1 intensity profile distribution was different from that of H5N1-derived eGFP-M1 and BFP-HA; the latter interaction was scattered diffusely in the cytoplasm without specific accumulation in the cell membrane (Fig 6B and 6C). mCherry-GNB1 specifically accumulated at the cell membrane with intensity profiles similar to those of H9N2 virus-derived eGFP-M1 and BFP-HA (Fig 6B and 6D).

**Fig 7:**
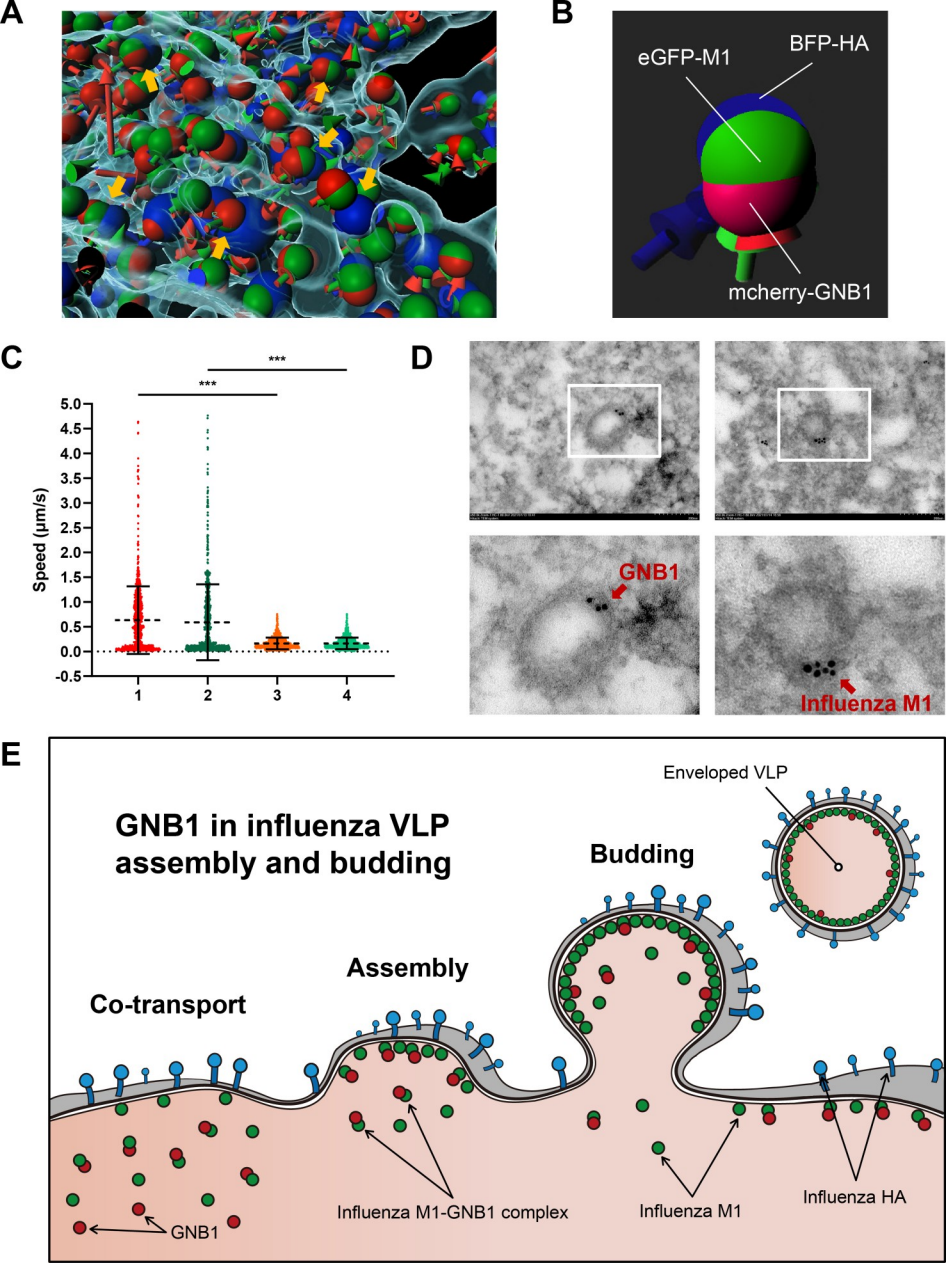
GNB1 protein co-transported with M1 protein in A549 cells. (A) A549 cells were transfected with *eGFP-M1*, *BFP-HA*, and *mCherry-GNB1* expression plasmids. Cross-section through the volume depicts the signal from the three channels. Yellow arrowheads indicate the co-localized puncta corresponding to co-transport of eGFP-M1 and mCherry-GNB1 to BFP-HA (S1 Movie). The blue, green, and red arrows or balls represent the tracks or punctas of BFP-HA, eGFP-M1, and mCherry-GNB1, respectively. (B) Enlarged view of the combination of H9N2-derived eGFP-M1, BFP-HA, and mCherry-GNB1. (C) Live-cell imaging illustrating the dynamic movement of the fluorescent fusion protein puncta within a cell with a similar number of spots and tracks between H9N2-derived *eGFP-M1/BFP-HA/mCherry-GNB1* and H5N1-derived *eGFP-M1/BFP-HA/mCherry-GNB1* transfected cells, which quantifies the track speed (ratio of track length to track duration [μm/s]) to characterize the intracellular movement. Each datapoint represents a punctum from the corresponding channel for the motion parameters (track speed) of eGFP-M1 singletons and mCherry-GNB1 singletons. “1” and “2” denote the speed of mCherry-GNB1 and H9N2-derived eGFP-M1 in H9N2-derived eGFP-M1/BFP-HA/mCherry-GNB1, respectively. “3” and “4” denote the speed of mCherry-GNB1 and H5N1-derived eGFP-M1 in H5N1-derived eGFP-M1/BFP-HA/mCherry-GNB1, respectively. (D) IEM analysis of influenza virus in the supernatant from infected cells. A549 cells were infected with rM14:M-H9N2 virus at an MOI of 1. At 24 hpi, supernatants from infected cells were collected for IEM. Anti-GNB1 or anti-influenza M1 antibody were used as the primary antibody. The gold is 10 nm in diameter. (E) Proposed model of the roles of GNB1 in viral assembly and budding of influenza VLPs.

Transfection of tagged viral and host proteins enables visualization of co-transport of virus protein and host component[46,47]. To further examine the dynamics of viral M1 and human GNB1 proteins, we performed live imaging of A549 cells transfected with *eGFP-M1* (H9N2- or H5N1-derived *M1*), *BFP-HA*, and *mCherry-GNB1*. The corresponding overlap of the three signals indicates similar trajectories (Fig 7A), and positional relationship (Fig 7B) between eGFP-M1, BFP-HA, and mCherry-GNB1 within the cell (S1 Movie). The speed of mCherry-GNB1 and eGFP-M1 proteins in H9N2 virus-derived *eGFP-M1*/*BFP-HA*/*mCherry-GNB1* transfected cells showed similar distribution with approximately equal means, which were significantly higher than that in H5N1-derived *eGFP-M1*/*BFP-HA*/*mCherry-GNB1* transfected cells (*P* < 0.001) (Fig 7C); this suggests that GNB1 facilitates the transport of H9N2 virus-derived M1. Furthermore, our results showed that both H9N2-derived M1 triggered VLPs and H5N1-derived M1 triggered VLPs could bud from the plasma membrane where mCherry-GNB1, BFP-HA, and eGFP-M1 clustered. However, more H9N2-derived M1 triggered VLPs accumulated at the plasma membrane (S5 Fig), suggesting that GNB1 assists the transport of H9N2 virus-derived M1 and assembly of the influenza VLPs. To further confirm this finding, we performed IEM. As shown in Fig 7D, GNB1 was detected in influenza virions. These results indicate that specific interaction between the H9N2 virus-derived M1 protein and the human GNB1 protein promotes transport of M1 protein to the cell membrane in mammalian cells, which in turn facilitates the binding of M1 and HA, and enhances the budding of H5N6 viral particles (a proposed model is shown in Fig 7E).

### GNB1 protein contributed to viral particle release triggered by H9N2 virus-derived M1 protein

**Fig 8:**
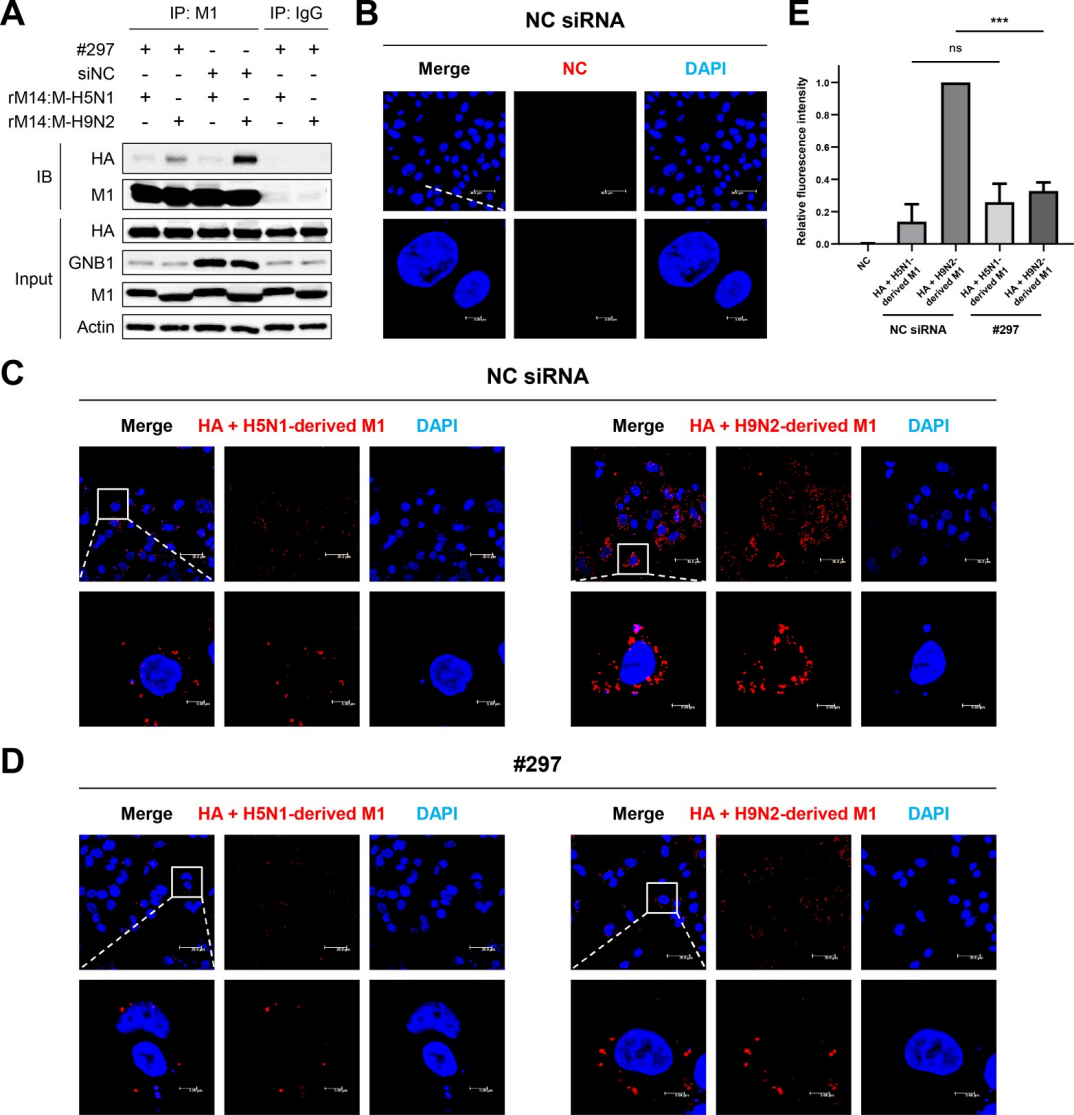
GNB1 facilitated interaction between M1 and HA proteins in mammalian cells. (A) A549 cells were transfected with NC siRNA or #297 siRNA and infected with recombinant H5N6 virus (rM14:M-H5N1 or rM14:M-H9N2). At 24 hpi, cell lysates were immunoprecipitated using an anti-influenza M1 antibody and probed with an anti-influenza HA antibody. Cell lysates of infected A549 cells were immunoprecipitated using an anti-IgG antibody and probed with an anti-influenza HA antibody as negative control. Compared to the normal cells, the interaction between H9N2-derived M1 and HA proteins was decreased in the GNB1-silenced cells. IB, immunoblot. (B–D) Influenza M1 and HA proteins interaction was determined by PLA. The PLA was performed using antibodies specific to influenza M1 and HA proteins. The fluorescence of cells was analyzed by a fluorescence confocal microscope (red fluorescent signal). Nuclei were stained with DAPI (blue). (B) PLA of the cells transfected with NC siRNA and an empty vector. (C) PLA of the cells transfected with NC siRNA, HA, and H9N2- or H5N1-derived M1 encoding plasmids. (D) PLA of the cells transfected with #297 siRNA, HA, and H9N2- or H5N1-derived M1 encoding plasmids. (E) Multiple images (B–D) were processed by BlobFinder to measure the PLA fluorescence intensity per cell (~30 cells total for each condition). The graphs show the means ± SD of three independent experiments normalized to the HA+H9N2-derived M1 (NC siRNA) PLA signal. (***, *P* < 0.001; ns, no significance.)

To determine whether GNB1 in assisting transport of H9N2 virus-derived M1 protein to the cell membrane could influence budding of VLPs, we knocked down the expression of *GNB1* gene in A549 cells by transfection with #297 siRNA to monitor the interaction between HA and M1 proteins. *GNB1* knockdown decreased binding between H9N2 virus-derived M1 and HA proteins in Co-IP assays (Fig 8A). The interaction between M1 and GNB1 proteins was determined by PLA in A549 cells. In *GNB1*-knockdown cells transfected with H9N2 virus-derived *M1* and *HA*, fluorescence intensity of PLA signals was significantly reduced (*P* < 0.001) (Fig 8B–8E). Collectively, these results confirm that GNB1 protein promotes the interaction of H9N2 virus-derived M1 and HA proteins in mammalian cells.

**Fig 9:**
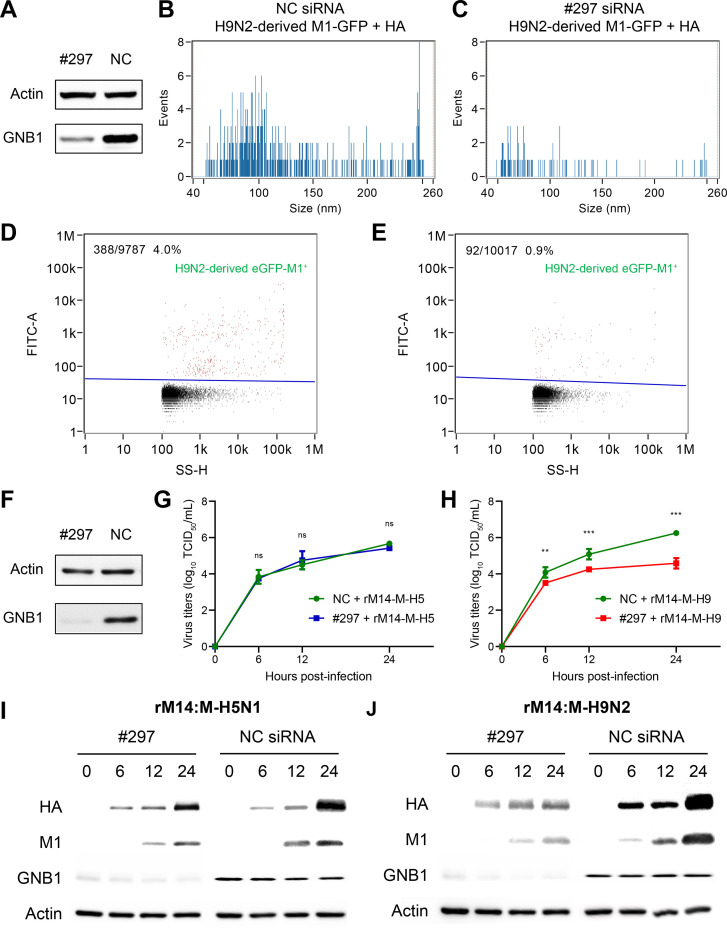
GNB1 protein contributed to viral particle release triggered by H9N2 virus-derived M1 protein. (A–E) Sizing and molecular profiling of VLP-associated H9N2-derived eGFP-M1 in #297 siRNA-induced GNB1-silenced or normal 293T cells. (A) A549 cells were transfected with siRNAs (targeting GNB1 or negative control), and GNB1 protein expression level at 36 h post-transfection were determined by Western blotting. (B–C) Size profiling of H9N2-derived eGFP-M1^+^ VLP particles with the FITC channel. (D–E) Molecular profiling of H9N2-derived eGFP-M1^+^ VLP particles. SS-H, side scatter height. (F–J) GNB1 knockdown decreased rM14:M-H9N2 virus replication in A549 cells. (F) GNB1 protein expression level at 36 h post-transfection determined by Western blotting. A549 cells transfected with siRNAs (either NC or GNB1-targeting siRNA #297) were infected with (G) rM14:M-H5N1 virus or (H) rM14:M-H9N2 virus at an MOI of 1. Viral titers were measured by TCID_50_ assay at the indicated time points. Each data is represented as means ± SD and represents three independent experiments. (**, *P* < 0.01; ***, *P* < 0.001; ns, no significance). A549 cells transfected with NC or GNB1-targeting siRNA #297 were infected with (I) rM14:M-H5N1 virus or(J) rM14:M-H9N2 virus at an MOI of 1, and expression of HA and M1 protein was measured by Western blotting.

To investigate the role of GNB1 in the assembly and release of influenza viral particles, 293T cells (with and without *GNB1* knockdown) were transfected with H9N2 virus-derived *eGFP-M1* and *HA* gene (Fig 9A). NanoFCM were performed to quantify the secreted H9N2 virus-derived eGFP-M1^+^ EVs. The main peak size of EVs of H9N2 virus-derived eGFP-M1^+^ particles was approximately 100 nm (Fig 9B). The percentage of H9N2 virus-derived eGFP-M1^+^ particles in the NC siRNA-transfected group was 4.0% (Fig 9D), whereas H9N2 virus-derived eGFP-M1^+^ particles in the GNB1-silenced group was 0.9% (Fig 9C and 9E). These results demonstrate that *GNB1* knockdown reduces the release of influenza VLPs.

To assess whether GNB1 affects viral replication, *GNB1*-knockdown A549 cells (Fig 9F) were separately infected with rM14:M-H5N1 and rM14:M-H9N2 virus (at MOI of 1.0). *GNB1* knockdown significantly decreased virus titers of rM14:M-H9N2 at 6, 12 and 24 hpi (*P* < 0.01) (Fig 9H), and inhibited the expression of viral HA and M1 proteins in a time-dependent manner at 6, 12 and 24 hpi (Fig 9J). However, *GNB1* knockdown did not affect the rM14:M-H5N1 virus replication in A549 cells (Fig 9G and 9I). Collectively, these data suggest that GNB1 is intimately involved in the replication cycle of H5N6 virus that encodes H9N2 virus-derived M1 protein.

## Discussion

H9N2 AIVs are a major threat to human public health by acting as donors of viral genes through reassortment with co-circulating influenza viruses. Here, we demonstrated that H9N2 virus-derived M1, but not H5N1 virus-derived M1, enhances interaction with human host factor GNB1, to promote M1 transport to the cell membrane which, in turn, facilitates the interaction between M1 and HA proteins, ultimately improving viral assembly and release. Our results suggest that H9N2 virus-derived M1 protein, along with surface HA, is key to increased incidence of H5N6 influenza virus infection in humans.

H9N2 and H5Ny AIVs have been circulating throughout the world, causing substantial economic losses to the poultry industry. In particular, these virus subtypes have been shown to infect humans[5,48]. A systematic review reported seroprevalence to H9N2 virus at 1–43% in avian-exposed human populations[49], and the seroprevalence of H9N2 antibodies in poultry workers in China was reported at 11.2%[50], suggesting significant infectivity of H9N2 AIVs in humans. Notably, H9N2 AIVs are regarded as “enablers” because they can donate their internal genes through reassortment to produce novel reassortants with higher human infectivity[7–12]. Given that the replacement with the G1-like *M* gene is a critical change in generating the G57 genotype of H9N2 influenza viruses[22], we focused on the contribution of the H9N2 virus-derived *M* gene to the replication of H5N6 virus in mammalian (human) cells. Influenza virus assembly postulate a direct interaction between the viral matrix protein M1 and the cytoplasmic domains of the envelope glycoproteins HA and/or NA as a driving force for virus assembly[23]. However, such an interaction has never been demonstrated biochemically. Here, we found that the H5N6 virus harboring the H9N2 virus-derived *M* gene, but not the H5N1-derived *M* gene, conferred higher binding affinity between M1 and HA proteins, which leads to increased budding of viral progeny in mammalian cells. Although we identify that C-terminal region of M1 protein is a key functional domain affecting the interaction with HA protein, further study is needed to determine the exact interaction sites that affect virus budding.

Multiple host proteins are involved in the assembly and budding of influenza virions and form part of a progeny particle[39,40]. GNB1 is the beta subunit of the family of G proteins, involved in regulating transmembrane signal transduction[51]. G proteins can be transferred between cells and are involved in apical versus basolateral transport in polarized epithelial cells, such as the apical transport of HA protein in virus-infected MDCK cells[52,53]. Initiation of virus budding may involve processes similar to membrane ruffling that are affected by cytoskeletal components, lipid rafts and signal transduction mediated by G proteins[54]. Thus, G protein-targeting drugs could conceivably inhibit virus budding[44]. It is believed that G proteins participate in the replication cycle of AIVs, wherein viral proteins interact directly or indirectly with G proteins[44,55,56]. However, the precise role of the G protein in influenza virus budding hitherto has remained unclear[44]. Here, we demonstrated that GNB1 promotes the interaction of influenza HA and M1 proteins during viral assembly to enhance progeny virus release.

Most studies to date have focused on the nucleocytoplasmic shuttling of vRNP, whilst only a few studies have investigated the transport of viral proteins to the plasma membrane[47]. In our study, using live-cell imaging, we found that host GNB1, in specific concert with H9N2 virus-derived M1, exerts a boosting effect on virus budding, which involves GNB1 binding of M1 and directing it to the viral assembly and budding site at the cell membrane. This mechanism would account in part for H9N2 virus-derived internal genes to be highly represented in human-derived avian H5N6 viruses. Our results reveal a key mechanism underlying AIVs infection in humans and provide a theoretical basis for a novel host-based antiviral target.

## Materials and methods

### Phylogenetic analyses

All available gene sequences of H5N6 AIVs in China were downloaded from the NCBI Influenza Virus Database (https://www.ncbi.nlm.nih.gov/genomes/FLU/) and the GISAID (https://www.gisaid.org/). RAxML V. 8.2.12 was used to construct maximum likelihood phylogenies for each gene segment[33] via the CIPRES Science Gateway V. 3.3[57]. One thousand bootstrap replicates were run, and GTRGAMMA + I was used as the nucleotide substitution model. Each internal gene was classified based on the evolutionary lineage. Based on phylogenetic analyses, the internal genes of H5N6 viruses were classified into different lineages according to the tree topology and bootstrap values (> 70).

### Cells and viruses

293T cells, Calu-3 cells, A549 cells, MDCK cells and DF-1 cells were cultured in Dulbecco’s modified Eagle’s medium (DMEM; Gibco) supplemented with 10% fetal bovine serum (FBS; Gibco), 100 units/mL penicillin and 100 μg/mL streptomycin. All cells were cultured at 37°C in a humidified incubator with 5% CO_2_.

Clade 2.3.4.4 H5N6 HPAIV A/goose/Northern China/M14/2016 (M14) and A/goose/Northern China/M10/2016 (M10) and H3N2 human seasonal virus A/Kansas/14/2017 were propagated in 9 days SPF embryonated chicken eggs and stored at -80°C. M14 and M10 house the H9N2 virus-derived *M* gene and the H5N1-derived *M* gene, respectively. All experiments with H5 subtype viruses were performed in a biosafety level 3 containment.

### Generation of recombinant H5N6 viruses

All eight gene segments of the M14 virus (H9N2 virus-derived *M* gene) and the *M* gene of M10 (H5N1-derived *M* gene), amplified by reverse transcription-PCR (RT-PCR), were individually cloned into a dual-promoter plasmid pHW2000, as described previously[58]. The reverse genetic virus rM14:M-H9N2, carrying all eight genes from the M14 virus, and the reassortant virus rM14:M-H5N1, with the *M* gene from the M10 virus and the remaining seven genes from the M14 virus, were generated by reverse genetics in 293T cells. All viruses were propagated in 9-day-old SPF chicken embryos and sequences were verified before use.

### Virus titration and replication kinetics

TCID_50_ was determined in MDCK cells infected with 10-fold serially diluted viruses and cultured at 37°C in a 5% CO_2_ atmosphere for 48 h. Virus infected cells were examined by detection of viral NP using immunofluorescence. TCID_50_ value was calculated using the Reed-Muench method[59]. Multistep replication kinetics were determined by inoculating DF-1, MDCK, A549 and Calu-3 cells with viruses at an MOI of 0.01. Following 1 h incubation at 37°C, the cells were washed three times and incubated in serum-free DMEM. Supernatants were collected at 6, 12, 24, 36, 48, 60 and 72 hpi and titrated on MDCK cells. Each experiment was conducted three times, and each experiment was performed in triplicate.

### Antibodies and reagents

Anti-IAV M1 antibody was kindly provided by Prof. Wenjun Liu, Chinese Academy of Sciences. Anti-H5N1 IAV HA antibody (11689-T54) was from Sino Biological. Anti-GNB1 antibody (10247-2-AP) was from Proteintech. Anti-β-actin antibody was from Beyotime. Anti-IAV NP antibody (ab20343) and anti-karyopherin subunit beta 1 antibody (KPNB1, ab2811) were from Abcam. Anti-junction plakoglobin antibody (JUP, 2309) was from CST, anti-cleft lip and palate transmembrane protein 1 antibody (CLPTM1, sc-374619) was from Santa Technology.

### Colocalization detection by confocal microscope

DF-1 and A549 cells were infected with indicated viruses at an MOI of 1. Cells were fixed with 4% paraformaldehyde in phosphate-buffered saline (PBS) for 30 min and permeabilized with immunostaining permeabilization buffer with Triton X-100 (Beyotime) for 10 min. After blocking with 5% BSA in PBS, anti-IAV M1 or anti-H5N1 IAV HA antibodies were used to detect M1 or HA proteins. Next, the cells were washed three times with PBS and incubated with fluorescein isothiocyanate (FITC; KPL) or tetramethyl rhodamine isothiocyanate (TRITC; KPL) labeled secondary antibodies for 1 h at 37°C. Subsequently, the cells were washed three times with PBST, and images were acquired using Nikon A1 confocal microscope (Nikon).

### Western blotting

Western blotting was performed as previously described[60]. Briefly, cell lysates were prepared from transfected cells using radioimmunoprecipitation assay (RIPA) lysis buffer, heated at 100°C for 10 min and were separated by 10% sodium dodecyl sulfate-polyacrylamide gel electrophoresis. After electrophoresis, protein samples were electroblotted onto polyvinylidene difluoride (PVDF) membranes (Bio-Rad) and blocked for 2 h in Tris-buffered saline (10 mM Tris-HCl, pH 8.0, containing 150 mM NaCl) containing 5% (w/v) non-fat dry milk and 0.5‰ (v/v) Tween-20. The membranes were incubated with the primary antibodies overnight at 4°C, followed by incubated with corresponding horseradish peroxidase (HRP)-conjugated secondary antibodies for 1 h at room temperature. The presence of HRP was detected by using a Western Lightning chemiluminescence kit (Amersham, USA) according to the manufacturer’s protocol.

### Co-IP

For the Co-IP assays, cells were infected with the recombinant H5N6 virus (rM14:M-H5N1 or rM14:M-H9N2) at an MOI of 1. After 24 hpi, cells were washed with PBS and lysed in 450 μL cell lysis buffer for Western and IP containing protease and phosphatase inhibitors (Roche Life Science), and protein lysates were used for Co-IP. Lysates were incubated with anti-IAV M1 antibody overnight at 4°C, and then protein A/G agarose beads (Santa Cruz Biotechnology) were added to the samples for 3–4 h at 4°C. The beads were washed four times with cold lysis buffer and analyzed by Western blotting.

### In situ PLA microscopy

A DuoLink PLA kit (DUO92105-1KT, Sigma) was used to detect protein-protein interactions as described in the protocol. Briefly, cells were fixed, permeabilized, and incubated with corresponding primary antibodies diluted in DuoLink dilution buffer. After incubation with primary antibodies, cells were incubated with PLA probes at 37°C for 1 h, followed by 30 min ligation at 37°C, and 100 min amplification at 37°C. Finally, a detection solution consisting of fluorescently labeled oligonucleotides was added, and the labelled oligonucleotides were hybridized to the concatemeric products. Nuclei were stained with DAPI. The fluorescence of cells was analyzed by a fluorescence confocal microscope (SP8 Lightning; Leica Microsystems).

Images obtained from PLA experiments were processed using BlobFinder, which performs single-cell analysis and quantifies the fluorescence signal intensity per cell for each sample[61]. The signal intensity of at least 30 cells was averaged in each experiment. Additionally, the relative fluorescence intensity of three independent experiments was averaged and plotted.

### Bimolecular fluorescence complementation (BiFC)

For the construction of BiFC plasmids, the complementary DNAs (cDNAs) of M1 and HA were amplified by PCR. Sequences encoding the amino [residues 1–173 (VN)] of the Venus fluorescence protein were fused to the *HA* of H5N6 virus M14 which were designated as VN-HA-M14. The carboxyl [residues 155–228 (VC)] fragments of the Venus fluorescence protein were fused to the total *M1* of M14 termed VC-M1-M14. The VC fragments fused to the truncated M1 protein (containing either 1–87, 88–164 or 165–252 residues) of M14 were designated as VC-M1N-M14, VC-M1M-M14 and VC-M1C-M14, respectively. Each full-length or truncated VC-M1 plasmid was co-transfected with VN-HA-M14 into 293T cells. 24 h post-transfection, the fluorescence signals were analyzed.

### IEM

For IEM, the supernatant from transfected or infected cells were centrifuged at 300 ×*g* for 10 min at 4°C to remove cells and 10,000 ×*g* for 30 min to remove debris. Supernatant were ultracentrifuged at 120,000 ×*g* for 70 min. The precipitant was re-suspended in PBS, loaded onto a 30–60% sucrose density gradient, and centrifuged at 150,000 ×*g* in an SW40 rotor (Beckman) for 22 h at 4°C. After ultracentrifugation, the 30–45% sucrose density gradient was extracted and centrifuged at 120,000 ×*g* for 70 min at 4°C. The precipitant re-suspended in PBS was processed further. Aliquots were adsorbed onto the carbon-coated copper grids and stained with 2% phosphotungstic acid (pH 7.2) for 120 s.

For immunogold labeling, the grids were incubated sequentially with blocking solution (1% BSA and 0.15% glycine in PBS), primary antibody diluted in the blocking solution, and immune-gold conjugate electron microscope goat anti-rabbit IgG H&L (10 nm gold; Abcam) or goat anti-mouse IgG H&L (10 nm gold; Boster Biological Technology) diluted in blocking solution, for 1 h each, and then washed with blocking solution. The grids were fixed with 2.5% glutaraldehyde for 5 min and stained with 2% uranyl acetate (for VLPs on the cell surface) or 2% phosphotungstic acid (for VLPs in the supernatant). Finally, the samples were examined in an HT-7800 electron microscope at 80 kV (Hitachi).

### Sizing and molecular profiling of individual EVs

To investigate the role of M1 protein (of different origins) in assembly and release of influenza viral particles, we fused H9N2- or H5N1-derived *M1* to eGFP-tagged expression plasmid (pCMV-C-EGFP, Beyotime), and fused influenza *HA* to the pRK5 plasmid by homologous recombination. eGFP-M1-loaded VLPs were assessed from expression of HA and eGFP-M1 (H9N2- or H5N1-derived M1) proteins in 293T cells. Cells were cultured in DMEM containing 10% serum in a 10 cm dish and grown to 80% confluence. The culture supernatant was removed and washed twice with PBS before the addition of Opti-MEM (Gibco). The culture supernatant was harvested at 24 h post-transfection for EVs purification. The supernatant was differentially centrifuged at 300, 2,000, 10,000 and 100,000 ×*g* for 10, 20, 30 and 17 min, respectively. For each centrifugation, the pellets were washed once with PBS and centrifuged again. The 100,000 ×*g* ultracentrifugation was performed using TLA120.2 (Beckman) and Superspin 630/17 (Thermo Scientific) at 4°C.

The size distribution analysis assumes that the sample has a comparable refractive index with the silica nanoparticles. The Silica Nanospheres Cocktail (S16M-Exo) is used as the size standard, and a calibration curve is constructed based on the sizes of the particles in S16M-Exo and their side scattering intensities. Using this calibration curve, every single particle side scattering intensity is converted into the corresponding particle size.

By setting a fixed number of events, samples were collected and analyzed for the fluorescence intensity of eGFP-M1^+^ particles. The pellet obtained after ultracentrifugation at 100,000 ×*g* for 17 min at 4°C was re-suspended in 100 μL PBS for further analysis with a Flow NanoAnalyzer model type N30E (NanoFCM) equipped with a laser (488 nm) and two channels (SSC, FITC channel).

### Mass spectrometry assays

A549 cells were infected with rM14:M-H9N2 virus (MOI of 1) for 24 h. Cell lysates were then harvested from infected cells using cell lysis buffer for Western blotting and IP (Beyotime). Next, H9N2 virus-derived M1 complexes in the cell lysates were purified using an anti-M1 antibody and analyzed by LC-MS. IgG immunoprecipitants from rM14:M-H9N2-infected samples were used as negative control in LC-MS analysis. Proteins identified in the M1 immunoprecipitants, but not in the IgG immunoprecipitants, were considered as M1-interacting proteins.

### LiTone LBS light-sheet imaging

To examine the colocalization dynamics between the viral M1 and HA proteins in the plasma membrane of A549 cells, observations were made on H9N2 virus-derived *eGFP-M1/BFP-HA* and H5N1 virus-derived *eGFP-M1/BFP-HA* transfected A549 cells at 24 h post-transfection. The plasma membrane was labeled with CellMask Plasma Membrane 647. Pearson’s coefficients were analyzed in colocalized volume between eGFP-M1 and BFP-HA located at the membrane in 36-time frames (1-time frame duration = 42.62 s). Light sheet imaging data were acquired in XYCZT order and have enough intensity dynamic range with higher bit depth (16 bit). The spatial resolution of each channel is 250 nm (X axis) * 250 nm (Y axis) * 350 nm (Z axis) which would be improved to half numerical of all directions by deconvolution. For colocalization analysis, we transform tiff format into ims. file to use ImarisColoc, which is a quantification tool based on voxels.

To investigate the colocalization of GNB1, influenza HA and M1 proteins during intracellular transport in mammalian cells, we fused H9N2- or H5N1-derived *M1* to the eGFP-tagged expression plasmid (pCMV-C-EGFP, Beyotime), influenza *HA* to the BFP-tagged expression plasmid (pCMV-C-BFP, Beyotime), and *GNB1* to the mCherry-tagged expression plasmid (pCMV-C-mCherry, Beyotime) by homologous recombination. A549 cells were transfected with *BFP-HA*, *mCherry-GNB1*, and *eGFP-M1* (H9N2- or H5N1-derived *M1*), carrying plasmids and seeded on 5 mm round glass coverslips (10^5^ cells) for high-resolution live imaging using light-sheet microscopy. The coverslips were cleaned using 75% ethanol and UV radiated before seeding. Before imaging, cells on the coverslip were stained with Cell Mask Plasma Membrane 647 (Invitrogen) for 15 min and washed by PBS twice. DMEM (15 mL) with 20 mM HEPES lacking phenol red was pre-heated to 37°C and placed in the imaging chamber. Three-dimensional (3D) live-cell imaging was performed on a high-resolution light-sheet microscope (LiTone LBS Light-sheet Microscope; Light Innovation Technology) with a 350 nm ultra-thin beam aligned in advance. Coverslips seeded with cells were clamped on the sample holder and immediately inserted into the pre-heated imaging chamber filled with DMEM and stabilized at 37°C. The regions of interest were targeted in EPI fluorescence mode with a 10× objective lens, and captured in LBS mode using 25× water immersion objective lens (Nikon CFI75 25XW, N.A. = 1.1) and a 2.5 magnification zoom module with minimum laser excitations at 405, 488, 561, and 647 nm, respectively. Real-time protein dynamics were recorded as a time-lapse of z-stacks with 250 nm step-size for 4 h at 50-time points. The deconvolution (using a modified Richardson–Lucy deconvolution algorithm) and final 3D movies were generated using the 3D projection function provided by LitScan 2.0.2 software. To examine the co-transport of influenza M1, influenza HA, and GNB1, A549 cells were transfected with *eGFP-M1*, *BFP-HA*, and *mCherry-GNB1* and stained the cells with Cell Mask Plasma Membrane 647 (Invitrogen). Next, we performed four-color volumetric, live imaging of the fluorescent fusion protein transport mode within the same cell using the LBS Light-sheet and tracked eGFP-M1, BFP-HA, and mCherry-GNB1. The tracking workflow was developed by analyzing and distilling track data derived from 3D images. The LBS Light-sheet datasets feature isotropic resolution (< 350 nm) obtained at ~0.025 Hz volumetric temporal resolution to capture fast-moving molecules. The H9N2-derived eGFP-M1, BFP-HA, and mCherry-GNB1 channels overlap at a single time point for the same dataset. Spheres of corresponding colors depicted colocalized puncta from each species at that time point, while arrowheads of corresponding colors depicted their tracks over all time points. Significant co-transport between colocalized puncta was observed due to the proximity between individual track segments traveling along the same direction.

### siRNA-mediated gene knockdown

To knock down the indicated target gene *GNB1*, the chemically synthesized siRNA and negative control (NC) siRNA were obtained from GenePharma Company. NC means negative control showing a negative or null effect in GNB1 protein knockdown. A549 cells were transfected with 50 nM siRNA using 3μL Lipofectamine RNAiMAX (Invitrogen) for approximately 36 h, and then used for the subsequent analyses.

### Statistical analyses

All statistical analyses were performed using Prism V. 8.0.2 (GraphPad Software). Statistical significance was assessed using Student’s *t*-test, one-way ANOVA followed by a Dunnett post-hoc test, or Chi-square test. *P* < 0.05 was considered to indicate a statistically significant difference.

### Accession number(s)

The nucleotide sequences of the eight gene segments of influenza viruses of M14 and *M* gene of M10 are available from GenBank under accession numbers MW774363-MW774371.

## Supporting information

S1 Fig Phylogenetic trees for the *HA*, *NA*, *PB2*, *PB1*, *PA*, *NP*, *M* and *NS* genes of influenza viruses related to Fig 1. Phylogenetic trees were estimated using genetic distances calculated by maximum likelihood under the GTRGAMMA + I model. Scale bar is in units of nucleotide substitutions per site. Node labels represent bootstrap values. Virus labeled with a red asterisk were used in the present research.
(PDF)

S2 Fig Physical interaction of viral M1 and HA proteins in Calu-3 and MDCK cells. Calu-3 or MDCK cells were infected separately with rM14:M-H5N1 and rM14:M-H9N2 at an MOI of 0.01. At 24 hpi, cell lysates were immunoprecipitated with anti-M1 antibody or anti-IgG antibody, followed by Western blotting for influenza M1 and HA proteins. An increased binding ability were observed between H9N2-derived M1 and HA proteins than between H5N1-derived M1 and HA proteins in both Calu-3 and MDCK cells. IB, immunoblot.
(TIF)

S3 Fig Interaction of truncated H9N2 virus-derived M1 and HA proteins in 293T cells by BiFC assay. (A) Domain structure of M1 and schematic representation of truncated M1 BiFC constructs. Schematic diagram of the domain structure of M1 as defined by crystallography (coordinates are amino acid number) and the subdivisions used in this study. (B) Each truncated M1 plasmid was co-transfected with VN-HA-M14 plasmid into 293T cells. At 24 h post transfection, 293T cells were taken under the fluorescent microscope and light microscope successively. (C) The positive cell rate of 293T cells transfected with BiFC constructs. The positive cells were determined by the fluorescence signal at different groups after transfection. The positive cell rate was calculated by counting positive cell numbers in 500 cells at 3 fields. The values shown are means ± standard deviations of results for three independent experiments. Statistical significance was based on one-way ANOVA (***, *P* < 0.001).
(TIF)

S4 Fig Host protein interacting with H9N2 virus-derived M1 protein in A549 cells. (A) The Venn diagrams illustrate the number of candidate proteins interacts with M1 protein in A549 cells. (B) A549 cells were infected with the recombinant H5N6 virus (rM14:M-H5N1 or rM14:M-H9N2). At 24 hpi, cell lysates were immunoprecipitated with anti-M1 antibody and probed with anti-KPNB1, anti-JUP, anti-CLPTM1, and anti-GNB1 antibodies. Cell lysates extracted from infected A549 cells were immunoprecipitated with anti-IgG antibody and probed with anti-KPNB1, anti-JUP, anti-CLPTM1, and anti-GNB1 antibodies as negative control. KPNB1, JUP, CLPTM1, and GNB1 did not immunoprecipitate in the absence of M1 immunoprecipitation during virus infection, ruling out any artifacts caused by the anti-M1 antibody. Both H9N2- and H5N1-derived M1 proteins specifically bound to GNB1; however, H9N2 virus-derived M1 and GNB1 proteins exhibited higher affinity binding.
(TIF)

S5 Fig Longitudinal section depicts budding of VLPs from H9N2 virus-derived eGFP-M1, BFP-HA, mCherry-GNB1 and merged channels. The dashed line depicts the cell outline, while the solid line circle depicts the VLPs budding from the plasma membrane, and the triangle indicates the accumulation of eGFP-M1, BFP-HA, and mCherry-GNB1.
(TIF)

S1 Table Detailed information of human H5N6 viruses analyzed in this study. (DOCX)

S2 Table Sequence identity of the M14 virus compared with human-isolated H5N6 viruses possessing H9N2-derived internal genes. (DOCX)

S3 Table Host protein interacting with H9N2-virus derived M1 protein in mammal cell. (DOCX)

S1 Movie Image analysis workflow for spot identification and tracking. Sample dataset for live-imaging of A549 cells transfected with eGFP-M1, BFP-HA and mCherry-GNB1. For each time-lapse live-cell imaging dataset, blue, green and red arrows or balls represent the tracks or punctas of BFP-HA, eGFP-M1 and mCherry-GNB1. The cyan color indicates the cytoplasmic membrane.
(AVI)

S2 Movie Budding of VLPs from cell membrane in one stack. A time-lapse sample movie of numerous VLPs releasing from apical membranes in one stack (Z-start: 0.0 μm, Z-end: 50.0 μm, Z-step: 0.5000 μm) in A549 cells transfected with BFP-HA (blue), eGFP-M1 (green) and mCherry-GNB1 (red).
(MP4)
