# Supplementary material for: H9N2 virus-derived M1 protein promotes H5N6 virus release in mammalian cells: Mechanism of avian influenza virus inter-species infection in humans
Source: PLoS Pathog. 2021 Dec 3;17(12):e1010098. doi: 10.1371/journal.ppat.1010098 (PMC8641880; doi:10.1371/journal.ppat.1010098)
Supplement: S1 Fig — Phylogenetic trees were estimated using genetic distances calculated by maximum likelihood under the GTRGAMMA + I model. Scale bar is in units of nucleotide substitutions per site. Node labels represent bootstrap values. Virus labeled with a red asterisk were used in the present research. (PDF) [file ppat.1010098.s001.pdf]

HA

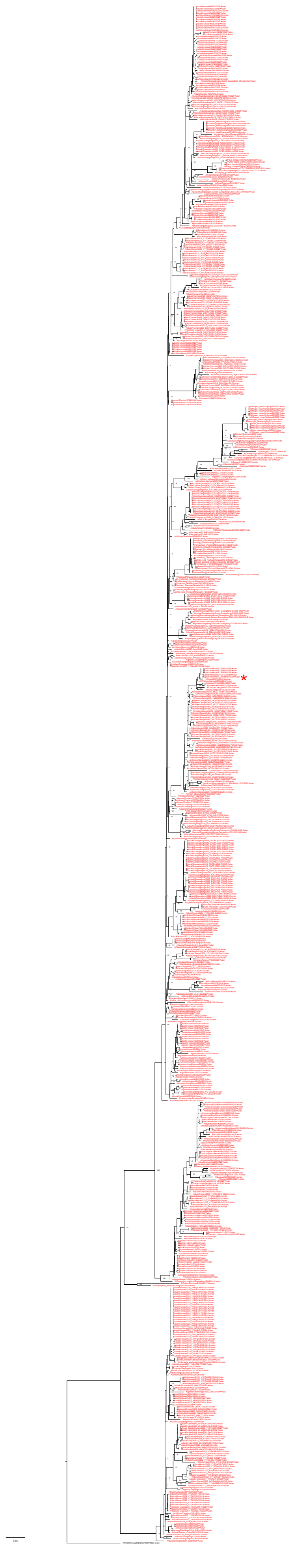

NA

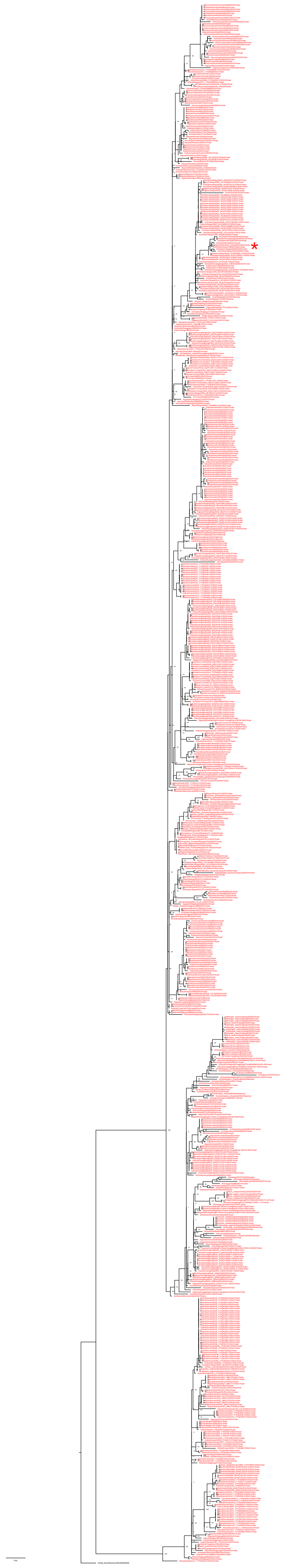

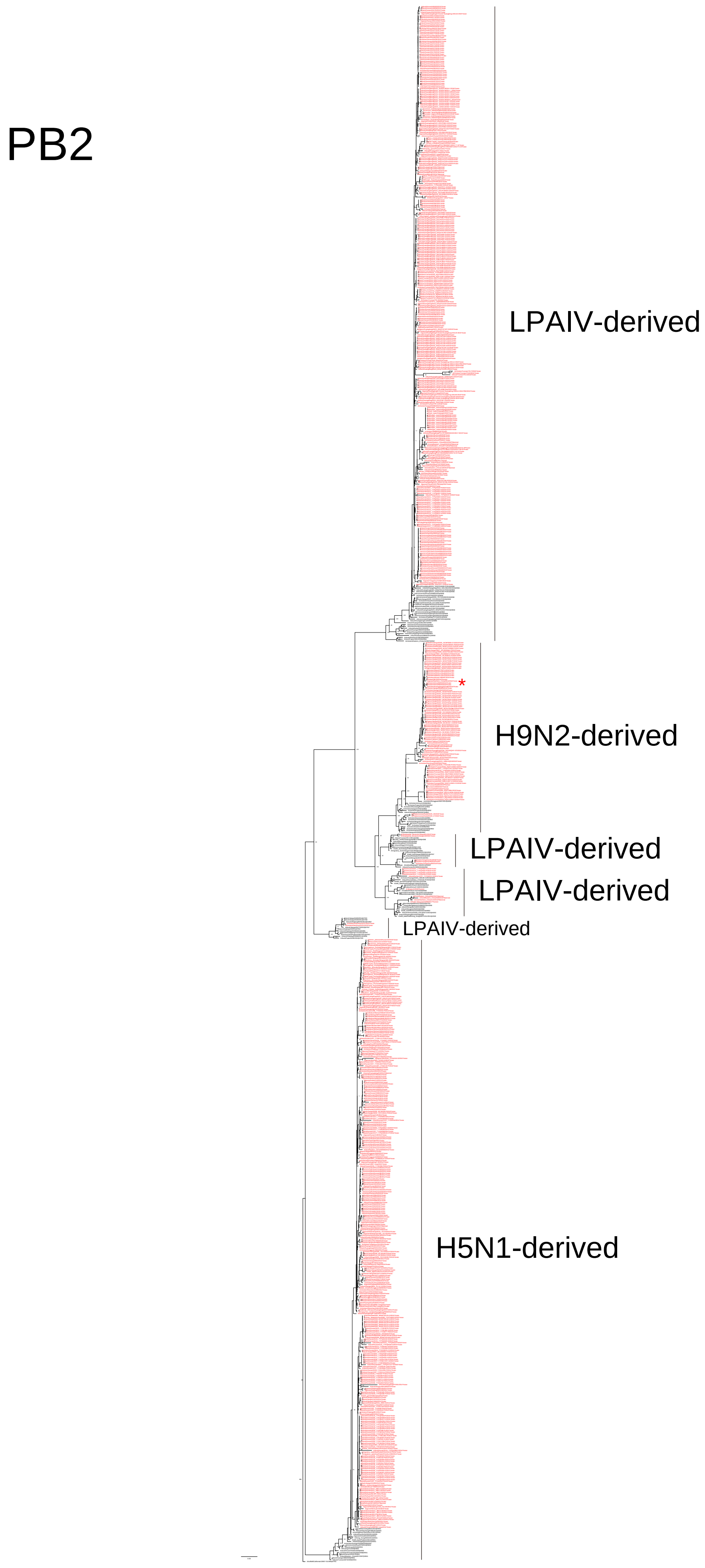
PB2
LPAIV-derived
*
H9N2-derived
LPAIV-derived
LPAIV-derived
LPAIV-derived
H5N1-derived

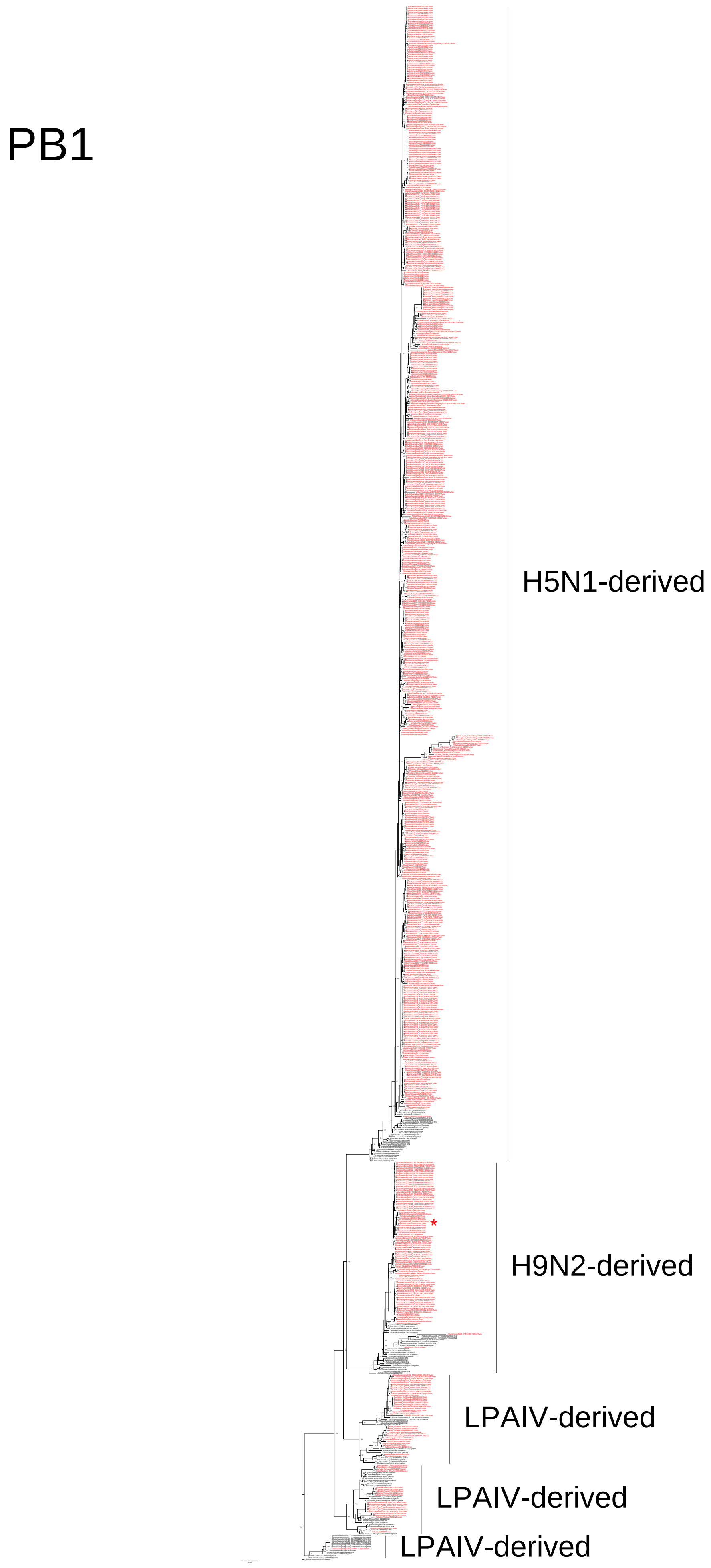
PB1
H5N1-derived
*
H9N2-derived
LPAIV-derived
LPAIV-derived
LPAIV-derived

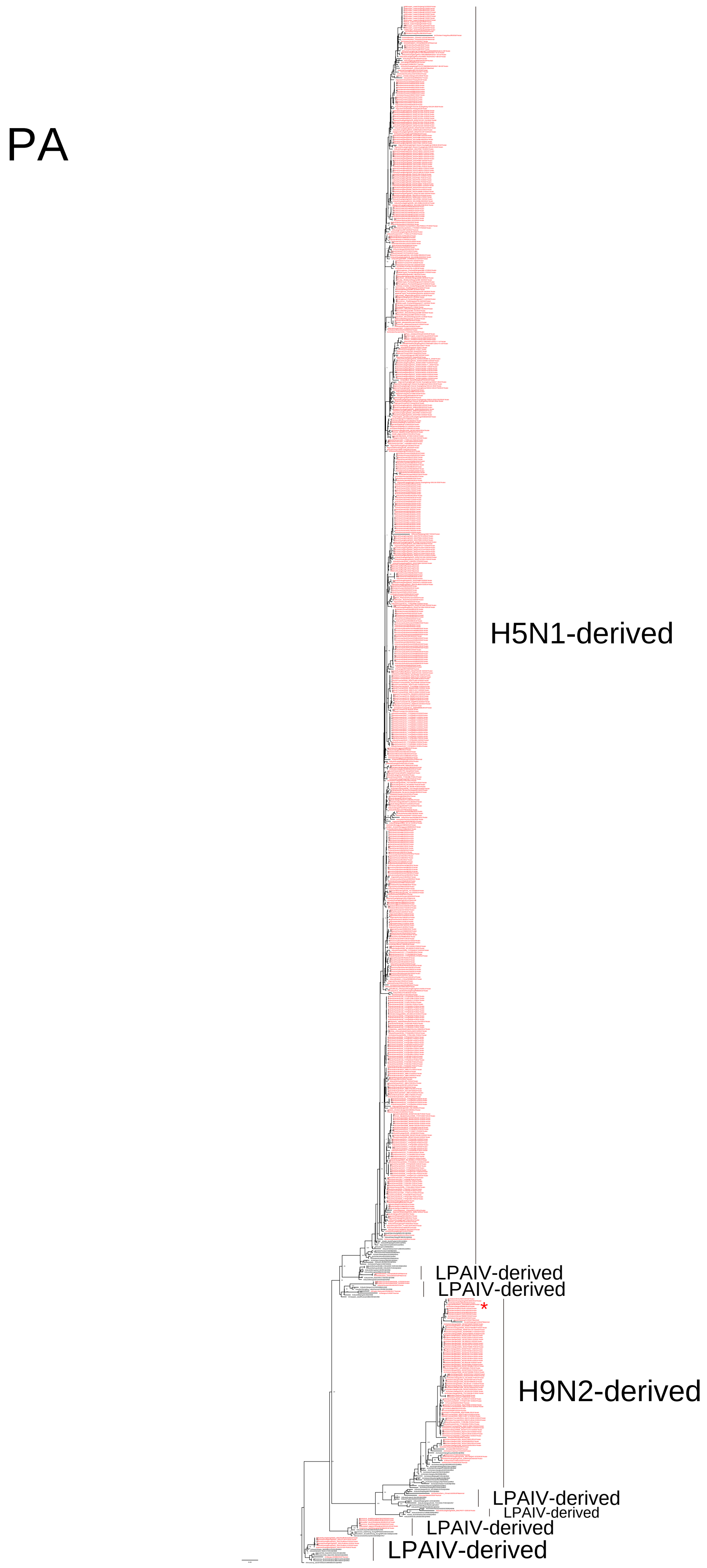
PA
H5N1-derived
LPAIV-derived
LPAIV-derived
*
H9N2-derived
LPAIV-derived
LPAIV-derived
LPAIV-derived
LPAIV-derived

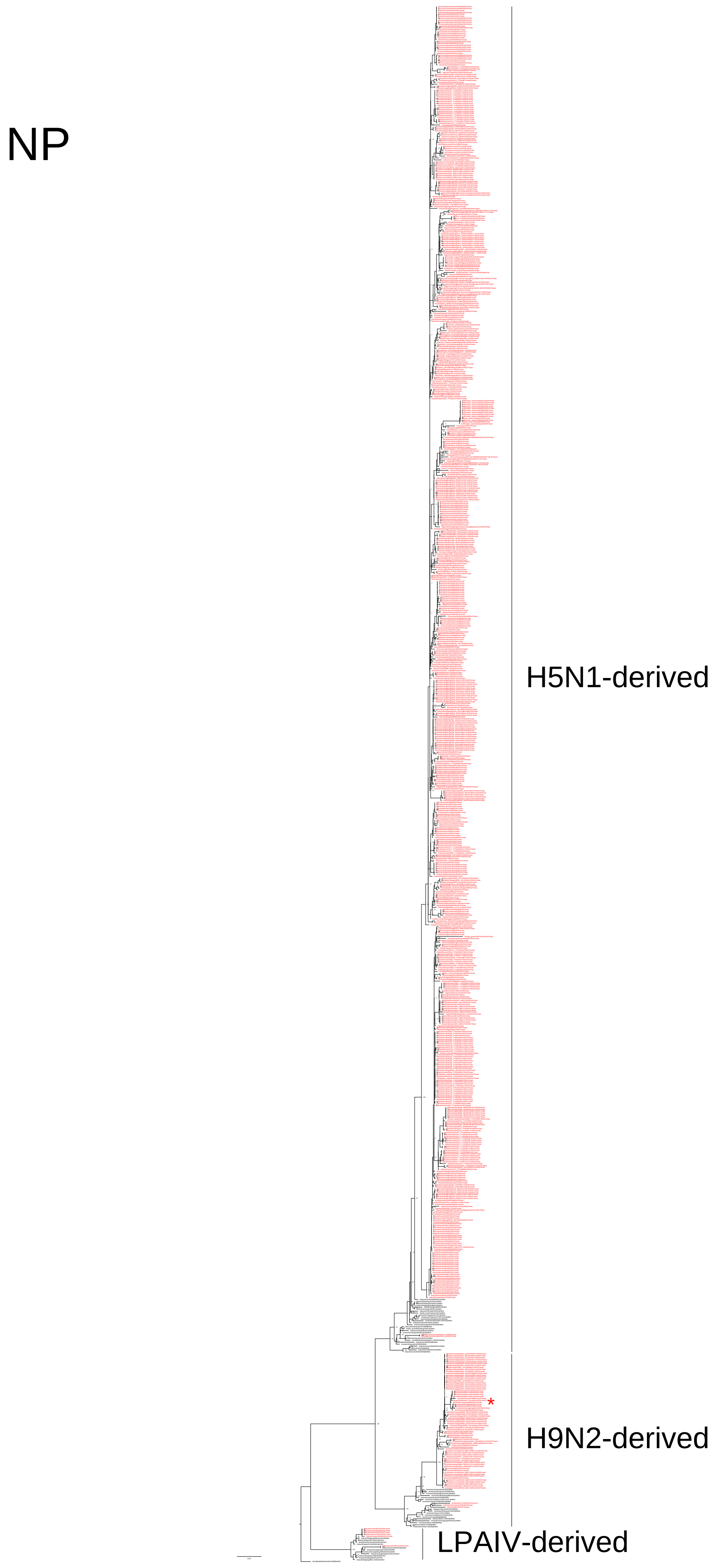
NP
H5N1-derived
H9N2-derived
LPAIV-derived

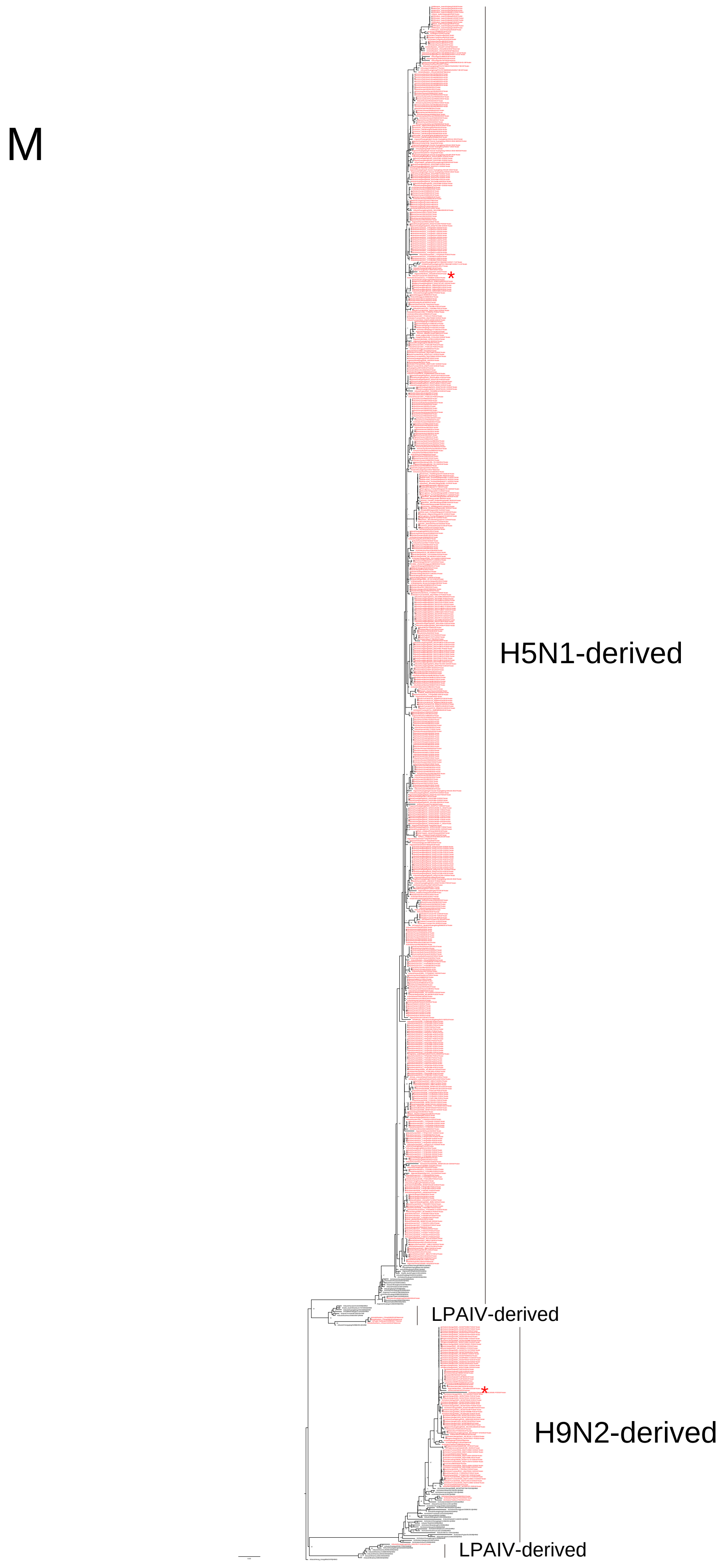
M
H5N1-derived
LPAIV-derived
H9N2-derived
LPAIV-derived

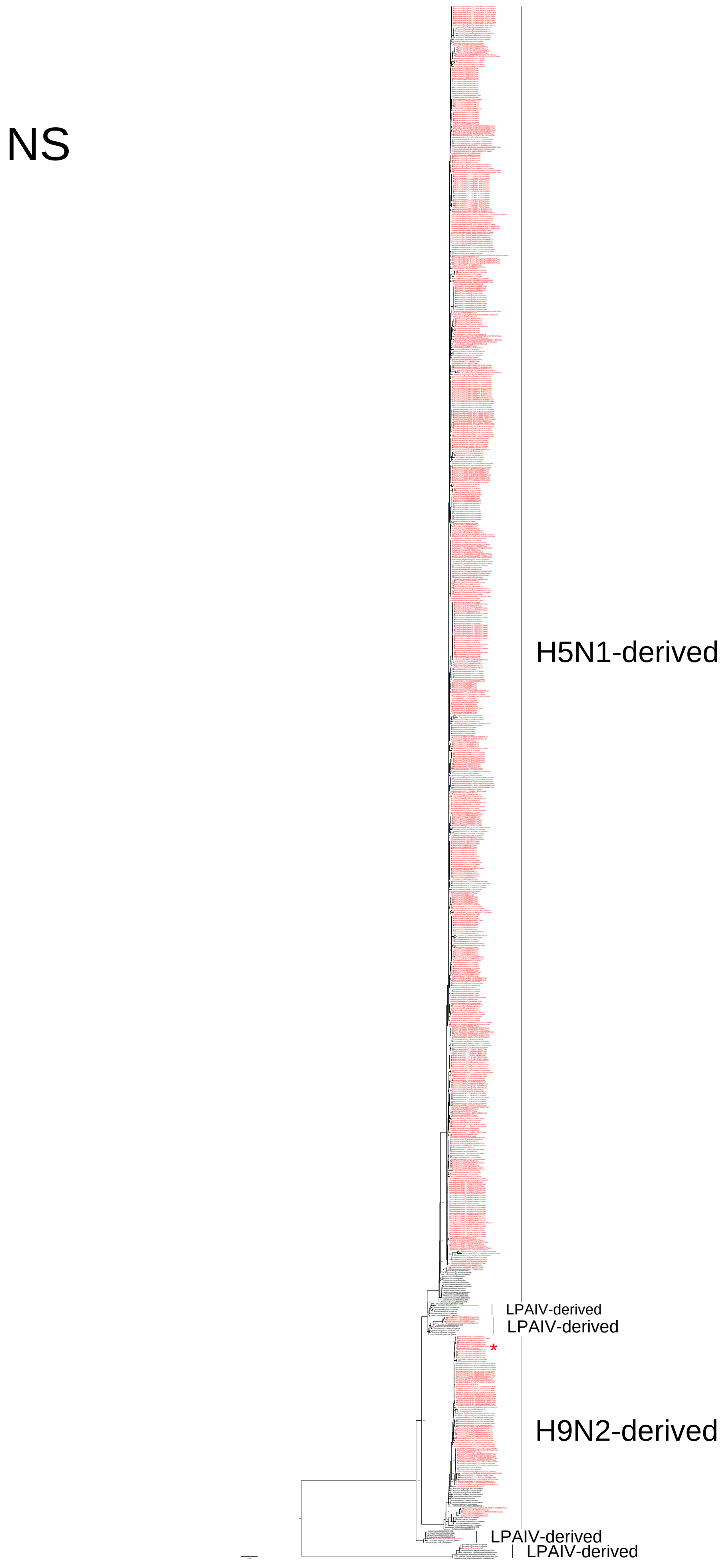
NS
H5N1-derived
LPAIV-derived
LPAIV-derived
*
H9N2-derived
LPAIV-derived
LPAIV-derived
