# Supplementary figures and images for: H9N2 virus-derived M1 protein promotes H5N6 virus release in mammalian cells: Mechanism of avian influenza virus inter-species infection in humans
Source: PLoS Pathog. 2021 Dec 3;17(12):e1010098. doi: 10.1371/journal.ppat.1010098 (PMC8641880; doi:10.1371/journal.ppat.1010098)

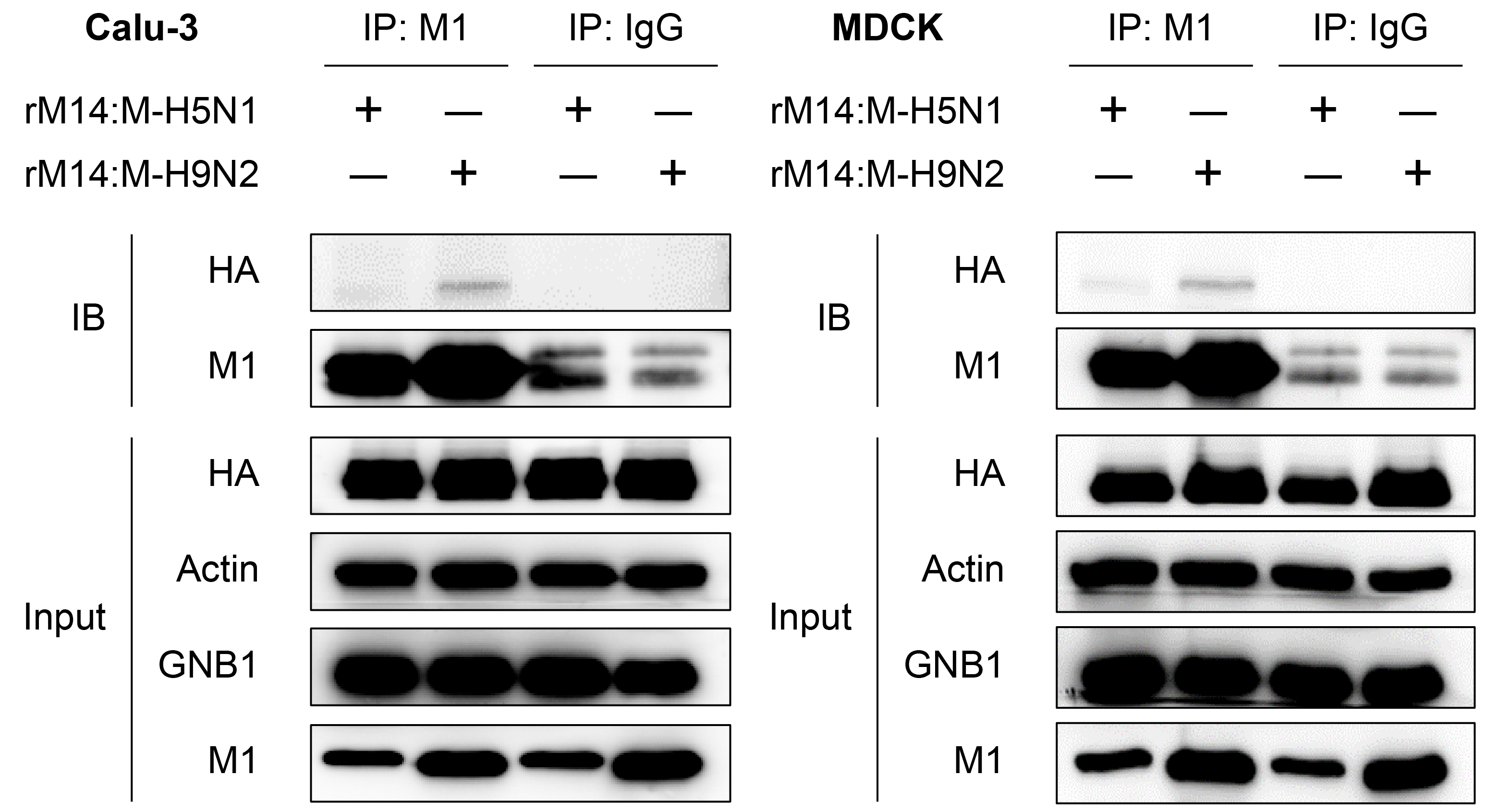

Supplement: S2 Fig — Calu-3 or MDCK cells were infected separately with rM14:M-H5N1 and rM14:M-H9N2 at an MOI of 0.01. At 24 hpi, cell lysates were immunoprecipitated with anti-M1 antibody or anti-IgG antibody, followed by Western blotting for influenza M1 and HA proteins. An increased binding ability were observed between H9N2-derived M1 and HA proteins than between H5N1-derived M1 and HA proteins in both Calu-3 and MDCK cells. IB, immunoblot. (TIF) [file ppat.1010098.s002.tif]

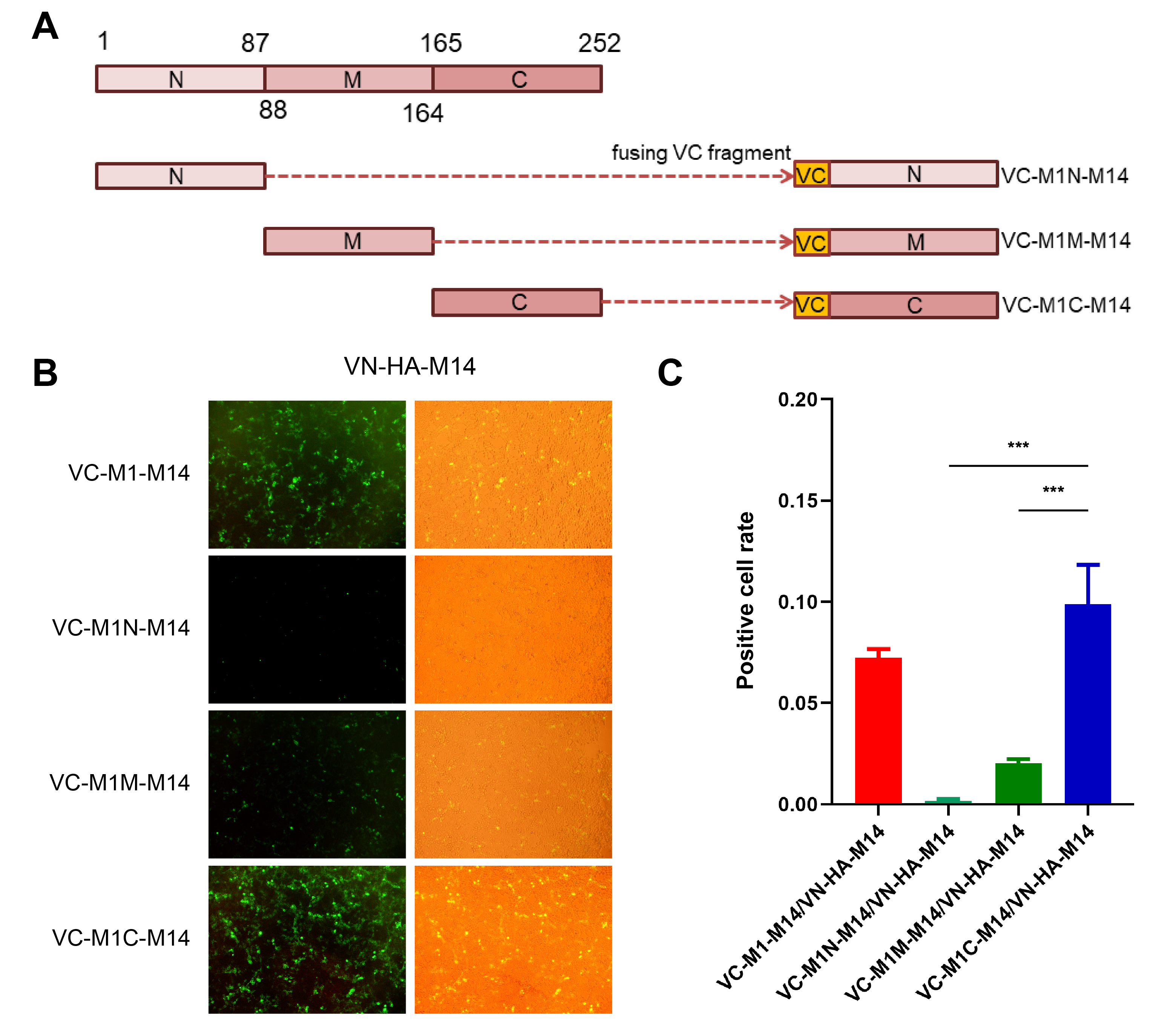

Supplement: S3 Fig — (A) Domain structure of M1 and schematic representation of truncated M1 BiFC constructs. Schematic diagram of the domain structure of M1 as defined by crystallography (coordinates are amino acid number) and the subdivisions used in this study. (B) Each truncated M1 plasmid was co-transfected with VN-HA-M14 plasmid into 293T cells. At 24 h post transfection, 293T cells were taken under the fluorescent microscope and light microscope successively. (C) The positive cell rate of 293T cells transfected with BiFC constructs. The positive cells were determined by the fluorescence signal at different groups after transfection. The positive cell rate was calculated by counting positive cell numbers in 500 cells at 3 fields. The values shown are means ± standard deviations of results for three independent experiments. Statistical significance was based on one-way ANOVA (***, P < 0.001). (TIF) [file ppat.1010098.s003.tif]

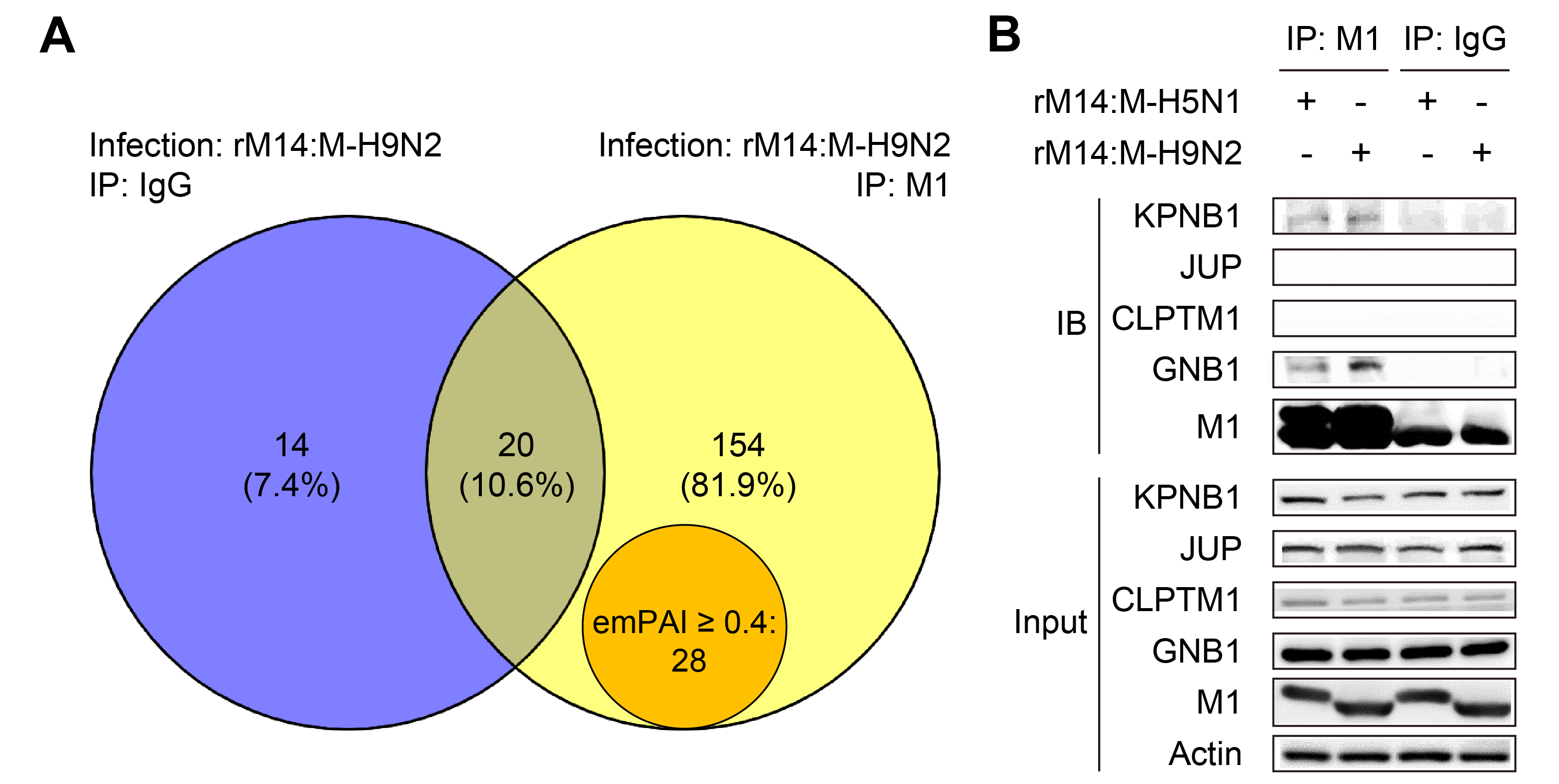

Supplement: S4 Fig — (A) The Venn diagrams illustrate the number of candidate proteins interacts with M1 protein in A549 cells. (B) A549 cells were infected with the recombinant H5N6 virus (rM14:M-H5N1 or rM14:M-H9N2). At 24 hpi, cell lysates were immunoprecipitated with anti-M1 antibody and probed with anti-KPNB1, anti-JUP, anti-CLPTM1, and anti-GNB1 antibodies. Cell lysates extracted from infected A549 cells were immunoprecipitated with anti-IgG antibody and probed with anti-KPNB1, anti-JUP, anti-CLPTM1, and anti-GNB1 antibodies as negative control. KPNB1, JUP, CLPTM1, and GNB1 did not immunoprecipitate in the absence of M1 immunoprecipitation during virus infection, ruling out any artifacts caused by the anti-M1 antibody. Both H9N2- and H5N1-derived M1 proteins specifically bound to GNB1; however, H9N2 virus-derived M1 and GNB1 proteins exhibited higher affinity binding. (TIF) [file ppat.1010098.s004.tif]

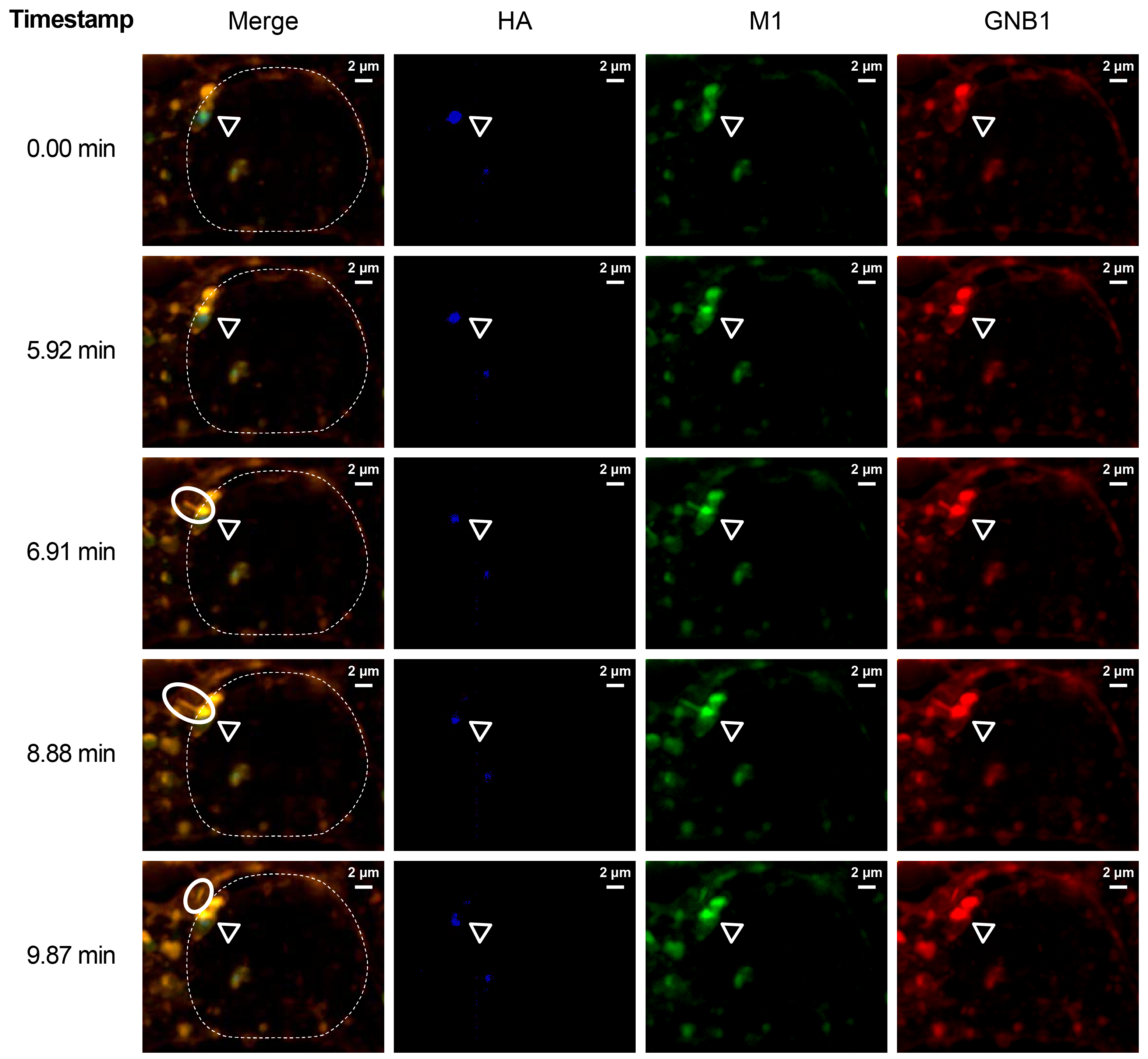

Supplement: S5 Fig — The dashed line depicts the cell outline, while the solid line circle depicts the VLPs budding from the plasma membrane, and the triangle indicates the accumulation of eGFP-M1, BFP-HA, and mCherry-GNB1. (TIF) [file ppat.1010098.s005.tif]
