# Supplementary material for: H9N2 virus-derived M1 protein promotes H5N6 virus release in mammalian cells: Mechanism of avian influenza virus inter-species infection in humans
Source: PLoS Pathog. 2021 Dec 3;17(12):e1010098. doi: 10.1371/journal.ppat.1010098 (PMC8641880; doi:10.1371/journal.ppat.1010098)
Supplement: S1 Table — (DOCX) [file ppat.1010098.s006.docx]

**S1 Table. Detailed information of human H5N6 viruses analyzed in this study.**

| **Strain name** | **Region** | **Collection year** | ***PB2*** | ***PB1*** | ***PA*** | ***NP*** | ***M*** | ***NS*** |
| --- | --- | --- | --- | --- | --- | --- | --- | --- |
| A/Guangzhou/39715/2014 | Guangzhou | 2014 | H5N1 | H5N1 | H5N1 | H5N1 | H5N1 | H5N1 |
| A/Sichuan/26221/2014 | Sichuan | 2014 | H5N1 | H5N1 | H5N1 | H5N1 | H5N1 | H5N1 |
| A/Guangdong/ZQ874/2015 | Guangdong | 2015 | Other LPAIV | H5N1 | H5N1 | H5N1 | H5N1 | H5N1 |
| A/Shenzhen/TH001/2015 | Shenzhen | 2015 | H9N2 | H9N2 | H9N2 | H9N2 | H9N2 | H9N2 |
| A/Yunnan/14563/2015 | Yunnan | 2015 | H9N2 | H9N2 | H9N2 | H9N2 | H9N2 | H9N2 |
| A/Yunnan/DQ001/2015 | Yunnan | 2015 | H9N2 | H9N2 | H9N2 | H9N2 | H9N2 | H9N2 |
| A/Yunnan/DQ002/2015 | Yunnan | 2015 | H9N2 | H9N2 | H9N2 | H9N2 | H9N2 | H9N2 |
| A/Anhui/33162/2016 | Anhui | 2016 | H9N2 | H9N2 | H9N2 | H9N2 | H9N2 | H9N2 |
| A/Guangxi/55726/2016 | Guangxi | 2016 | Other LPAIV | Other LPAIV | H5N1 | H5N1 | H5N1 | H5N1 |
| A/Hubei/29578/2016 | Hubei | 2016 | H9N2 | H9N2 | H9N2 | H9N2 | H9N2 | H9N2 |
| A/Hunan/55555/2016 | Hunan | 2016 | Other LPAIV | Other LPAIV | Other LPAIV | H5N1 | H5N1 | H5N1 |
| A/Shenzhen/TH002/2016 | Shenzhen | 2016 | H9N2 | H9N2 | H9N2 | H9N2 | H9N2 | H9N2 |
| A/Shenzhen/TH003/2016 | Shenzhen | 2016 | H9N2 | H9N2 | H9N2 | H9N2 | H9N2 | H9N2 |
| A/Fujian-Sanyuan/21099/2017 | Fujian | 2017 | Other LPAIV | Other LPAIV | Other LPAIV | H5N1 | Other LPAIV | Other LPAIV |
| A/Guangxi/13486/2017 | Guangxi | 2017 | Other LPAIV | H5N1 | H5N1 | H5N1 | H5N1 | H5N1 |
| A/Guangdong/18SF020/2018 | Guangdong | 2018 | Other LPAIV | H5N1 | H5N1 | H5N1 | H5N1 | H5N1 |
| A/Guangxi/31906/2018 | Guangxi | 2018 | Other LPAIV | H5N1 | H5N1 | H5N1 | H5N1 | H5N1 |
| A/Guangxi/32797/2018 | Guangxi | 2018 | Other LPAIV | H5N1 | H5N1 | H5N1 | H5N1 | H5N1 |
| A/Jiangsu/32888/2018 | Jiangsu | 2018 | Other LPAIV | H5N1 | Other LPAIV | H5N1 | H5N1 | H5N1 |
| A/Jiangsu/1/2020 | Jiangsu | 2020 | Other LPAIV | Other LPAIV | Other LPAIV | H5N1 | H5N1 | Other LPAIV |
