# Supplementary material for: H9N2 virus-derived M1 protein promotes H5N6 virus release in mammalian cells: Mechanism of avian influenza virus inter-species infection in humans
Source: PLoS Pathog. 2021 Dec 3;17(12):e1010098. doi: 10.1371/journal.ppat.1010098 (PMC8641880; doi:10.1371/journal.ppat.1010098)
Supplement: S2 Table — (DOCX) [file ppat.1010098.s007.docx]

**S2 Table. Sequence identity of the M14 virus compared with human-isolated H5N6 viruses possessing H9N2-derived internal genes.**

| **Strain name** | **Gene segment (%)^a^** | | | | | | | |  |
| --- | --- | --- | --- | --- | --- | --- | --- | --- | --- |
|  | ***PB2*** | ***PB1*** | ***PA*** | ***HA*** | ***NP*** | ***NA*** | ***M*** | ***NS*** |  |
| A/Yunnan/DQ001/2015 | 93.8 | 97.4 | 98.7 | 97.0 | 98.3 | 97.7 | 98.0 | 97.7 |  |
| A/Yunnan/DQ002/2015 | 93.7 | 97.4 | 98.2 | 96.7 | 98.4 | 97.3 | 97.9 | 97.7 |  |
| A/Yunnan/14563/2015 | 93.9 | 97.4 | 98.6 | 97.0 | 98.3 | 97.7 | 98.0 | 97.7 |  |
| A/Shenzhen/TH001/2015 | 96.7 | 97.0 | 95.8 | 98.4 | 97.9 | 98.8 | 98.8 | 94.4 |  |
| A/Shenzhen/TH002/2016 | 97.4 | 97.3 | 95.8 | 98.1 | 97.9 | 98.7 | 98.7 | 94.3 |  |
| A/Shenzhen/TH003/2016 | 97.5 | 97.1 | 96.1 | 97.9 | 94.1 | 98.8 | 98.4 | 97.1 |  |
| A/Hubei/29578/2016 | 99.6 | 95.5 | 95.8 | 99.4 | 94.5 | 99.8 | 99.7 | 99.9 |  |
| A/Anhui/33162/2016 | 98.0 | 97.4 | 97.7 | 98.2 | 98.2 | 97.8 | 99.1 | 99.4 |  |
| Mean ± SD | 96.3 ± 2.2 | 97.1 ± 0.7 | 97.1 ± 1.3 | 97.8 ± 0.9 | 97.2 ± 1.8 | 98.3 ± 0.8 | 98.6 ± 0.6 | 97.3 ± 2.0 |  |

^a^The percent identity was calculated based on coding sequence (CDS).
