# Supplementary material for: H9N2 virus-derived M1 protein promotes H5N6 virus release in mammalian cells: Mechanism of avian influenza virus inter-species infection in humans
Source: PLoS Pathog. 2021 Dec 3;17(12):e1010098. doi: 10.1371/journal.ppat.1010098 (PMC8641880; doi:10.1371/journal.ppat.1010098)
Supplement: S3 Table — (DOCX) [file ppat.1010098.s008.docx]

**S3 Table. Host protein interacting with H9N2-virus derived M1 protein in mammal cells.**

| **Family** | **Member** | **Database** | **Accession** | **Score** | **Mass** | **Num. of matches** | **Num. of significant matches** | **Num. of sequences** | **Num. of significant sequences** | **emPAI** | **Description** |
| --- | --- | --- | --- | --- | --- | --- | --- | --- | --- | --- | --- |
| 155 | 1 | uniprot_human | Q9UKZ4 | 16 | 309664 | 1 | 1 | 1 | 1 | 0.01 | sp\|Q9UKZ4\|TEN1_HUMAN Teneurin-1 OS=Homo sapiens GN=TENM1 PE=1 SV=2 |
| 139 | 1 | uniprot_human | E7EN95 | 20 | 258059 | 1 | 1 | 1 | 1 | 0.02 | tr\|E7EN95\|E7EN95_HUMAN Filamin-B OS=Homo sapiens GN=FLNB PE=1 SV=1 |
| 168 | 1 | uniprot_human | M0R0P8 | 15 | 244845 | 1 | 1 | 1 | 1 | 0.02 | tr\|M0R0P8\|M0R0P8_HUMAN Unconventional myosin-IXb OS=Homo sapiens GN=MYO9B PE=1 SV=1 |
| 62 | 1 | uniprot_human | Q8N139 | 41 | 185706 | 1 | 1 | 1 | 1 | 0.02 | sp\|Q8N139\|ABCA6_HUMAN ATP-binding cassette sub-family A member 6 OS=Homo sapiens GN=ABCA6 PE=1 SV=2 |
| 125 | 1 | uniprot_human | A0A0A0MS99 | 22 | 135813 | 1 | 1 | 1 | 1 | 0.03 | tr\|A0A0A0MS99\|A0A0A0MS99_HUMAN Multidrug resistance-associated protein 1 OS=Homo sapiens GN=ABCC1 PE=1 SV=1 |
| 150 | 1 | uniprot_human | G5E9Y3 | 18 | 152761 | 1 | 1 | 1 | 1 | 0.03 | tr\|G5E9Y3\|G5E9Y3_HUMAN Arf-GAP with Rho-GAP domain, ANK repeat and PH domain-containing protein 3 OS=Homo sapiens GN=ARAP3 PE=1 SV=1 |
| 145 | 1 | uniprot_human | H0YD14 | 18 | 135822 | 1 | 1 | 1 | 1 | 0.03 | tr\|H0YD14\|H0YD14_HUMAN Myoferlin (Fragment) OS=Homo sapiens GN=MYOF PE=1 SV=1 |
| 173 | 1 | uniprot_human | O14917 | 14 | 127405 | 1 | 1 | 1 | 1 | 0.03 | sp\|O14917\|PCD17_HUMAN Protocadherin-17 OS=Homo sapiens GN=PCDH17 PE=2 SV=2 |
| 67 | 1 | uniprot_human | P01023 | 36 | 164613 | 1 | 1 | 1 | 1 | 0.03 | sp\|P01023\|A2MG_HUMAN Alpha-2-macroglobulin OS=Homo sapiens GN=A2M PE=1 SV=3 |
| 135 | 1 | uniprot_human | P07814 | 20 | 172080 | 1 | 1 | 1 | 1 | 0.03 | sp\|P07814\|SYEP_HUMAN Bifunctional glutamate/proline--tRNA ligase OS=Homo sapiens GN=EPRS PE=1 SV=5 |
| 159 | 1 | uniprot_human | G3XAL9 | 16 | 125254 | 1 | 1 | 1 | 1 | 0.04 | tr\|G3XAL9\|G3XAL9_HUMAN Solute carrier family 12 (Sodium/potassium/chloride transporters), member 2, isoform CRA_a OS=Homo sapiens GN=SLC12A2 PE=1 SV=1 |
| 124 | 1 | uniprot_human | K4DI95 | 22 | 126257 | 3 | 3 | 1 | 1 | 0.04 | tr\|K4DI95\|K4DI95_HUMAN Diaphanous homolog 2 (Drosophila), isoform CRA_a OS=Homo sapiens GN=DIAPH2 PE=1 SV=1 |
| 160 | 1 | uniprot_human | Q08554 | 16 | 101406 | 1 | 1 | 1 | 1 | 0.04 | sp\|Q08554\|DSC1_HUMAN Desmocollin-1 OS=Homo sapiens GN=DSC1 PE=1 SV=2 |
| 103 | 1 | uniprot_human | Q2TB90 | 27 | 103790 | 1 | 1 | 1 | 1 | 0.04 | sp\|Q2TB90\|HKDC1_HUMAN Putative hexokinase HKDC1 OS=Homo sapiens GN=HKDC1 PE=1 SV=3 |
| 166 | 1 | uniprot_human | Q7Z2W4 | 15 | 103135 | 1 | 1 | 1 | 1 | 0.04 | sp\|Q7Z2W4\|ZCCHV_HUMAN Zinc finger CCCH-type antiviral protein 1 OS=Homo sapiens GN=ZC3HAV1 PE=1 SV=3 |
| 115 | 1 | uniprot_human | A0A0D9SF53 | 25 | 82110 | 1 | 1 | 1 | 1 | 0.05 | tr\|A0A0D9SF53\|A0A0D9SF53_HUMAN ATP-dependent RNA helicase DDX3X OS=Homo sapiens GN=DDX3X PE=1 SV=1 |
| 142 | 1 | uniprot_human | O95573 | 19 | 81338 | 1 | 1 | 1 | 1 | 0.05 | sp\|O95573\|ACSL3_HUMAN Long-chain-fatty-acid--CoA ligase 3 OS=Homo sapiens GN=ACSL3 PE=1 SV=3 |
| 50 | 1 | uniprot_human | P33993 | 51 | 81884 | 1 | 1 | 1 | 1 | 0.05 | sp\|P33993\|MCM7_HUMAN DNA replication licensing factor MCM7 OS=Homo sapiens GN=MCM7 PE=1 SV=4 |
| 89 | 1 | uniprot_human | Q9Y4W6 | 30 | 88984 | 1 | 1 | 1 | 1 | 0.05 | sp\|Q9Y4W6\|AFG32_HUMAN AFG3-like protein 2 OS=Homo sapiens GN=AFG3L2 PE=1 SV=2 |
| 123 | 1 | uniprot_human | E5RI94 | 22 | 73920 | 1 | 1 | 1 | 1 | 0.06 | tr\|E5RI94\|E5RI94_HUMAN TBC1 domain family member 31 OS=Homo sapiens GN=TBC1D31 PE=1 SV=1 |
| 112 | 1 | uniprot_human | H3BLZ8 | 25 | 81073 | 1 | 1 | 1 | 1 | 0.06 | tr\|H3BLZ8\|H3BLZ8_HUMAN Probable ATP-dependent RNA helicase DDX17 OS=Homo sapiens GN=DDX17 PE=1 SV=1 |
| 156 | 1 | uniprot_human | O43296 | 16 | 72594 | 1 | 1 | 1 | 1 | 0.06 | sp\|O43296\|ZN264_HUMAN Zinc finger protein 264 OS=Homo sapiens GN=ZNF264 PE=1 SV=1 |
| 73 | 1 | uniprot_human | P53621 | 30 | 139797 | 2 | 2 | 2 | 2 | 0.06 | sp\|P53621\|COPA_HUMAN Coatomer subunit alpha OS=Homo sapiens GN=COPA PE=1 SV=2 |
| 66 | 1 | uniprot_human | A0A087WWY3 | 37 | 248149 | 4 | 4 | 4 | 4 | 0.07 | tr\|A0A087WWY3\|A0A087WWY3_HUMAN Filamin-A OS=Homo sapiens GN=FLNA PE=1 SV=1 |
| 97 | 1 | uniprot_human | A0A0A0MT26 | 29 | 135170 | 2 | 2 | 2 | 2 | 0.07 | tr\|A0A0A0MT26\|A0A0A0MT26_HUMAN Sodium/potassium-transporting ATPase subunit alpha-3 OS=Homo sapiens GN=ATP1A3 PE=1 SV=1 |
| 128 | 1 | uniprot_human | G3V3B0 | 21 | 67683 | 1 | 1 | 1 | 1 | 0.07 | tr\|G3V3B0\|G3V3B0_HUMAN Apoptotic chromatin condensation inducer in the nucleus OS=Homo sapiens GN=ACIN1 PE=1 SV=1 |
| 172 | 1 | uniprot_human | H0YD68 | 15 | 61181 | 1 | 1 | 1 | 1 | 0.07 | tr\|H0YD68\|H0YD68_HUMAN Outer dense fiber protein 2-like (Fragment) OS=Homo sapiens GN=ODF2L PE=1 SV=1 |
| 80 | 1 | uniprot_human | J3KPF3 | 33 | 68230 | 1 | 1 | 1 | 1 | 0.07 | tr\|J3KPF3\|J3KPF3_HUMAN 4F2 cell-surface antigen heavy chain OS=Homo sapiens GN=SLC3A2 PE=1 SV=1 |
| 68 | 1 | uniprot_human | Q6UWP8 | 35 | 60562 | 2 | 2 | 1 | 1 | 0.07 | sp\|Q6UWP8\|SBSN_HUMAN Suprabasin OS=Homo sapiens GN=SBSN PE=1 SV=2 |
| 151 | 1 | uniprot_human | Q96II5 | 18 | 68681 | 1 | 1 | 1 | 1 | 0.07 | tr\|Q96II5\|Q96II5_HUMAN ARAF protein OS=Homo sapiens GN=ARAF PE=1 SV=1 |
| 122 | 1 | uniprot_human | Q9HD45 | 22 | 68584 | 1 | 1 | 1 | 1 | 0.07 | sp\|Q9HD45\|TM9S3_HUMAN Transmembrane 9 superfamily member 3 OS=Homo sapiens GN=TM9SF3 PE=1 SV=2 |
| 59 | 1 | uniprot_human | B4DUR8 | 42 | 56152 | 1 | 1 | 1 | 1 | 0.08 | tr\|B4DUR8\|B4DUR8_HUMAN T-complex protein 1 subunit gamma OS=Homo sapiens GN=CCT3 PE=1 SV=1 |
| 38 | 1 | uniprot_human | F5H5D3 | 70 | 58606 | 1 | 1 | 1 | 1 | 0.08 | tr\|F5H5D3\|F5H5D3_HUMAN Tubulin alpha-1C chain OS=Homo sapiens GN=TUBA1C PE=1 SV=1 |
| 76 | 1 | uniprot_human | P78347 | 33 | 112859 | 2 | 2 | 2 | 2 | 0.08 | sp\|P78347\|GTF2I_HUMAN General transcription factor II-I OS=Homo sapiens GN=GTF2I PE=1 SV=2 |
| 87 | 1 | uniprot_human | Q96SQ9 | 31 | 56009 | 1 | 1 | 1 | 1 | 0.08 | sp\|Q96SQ9\|CP2S1_HUMAN Cytochrome P450 2S1 OS=Homo sapiens GN=CYP2S1 PE=1 SV=2 |
| 111 | 1 | uniprot_human | Q9UPY5 | 26 | 55786 | 1 | 1 | 1 | 1 | 0.08 | sp\|Q9UPY5\|XCT_HUMAN Cystine/glutamate transporter OS=Homo sapiens GN=SLC7A11 PE=1 SV=1 |
| 61 | 1 | uniprot_human | B0YJC4 | 41 | 49680 | 1 | 1 | 1 | 1 | 0.09 | tr\|B0YJC4\|B0YJC4_HUMAN Vimentin OS=Homo sapiens GN=VIM PE=1 SV=1 |
| 49 | 1 | uniprot_human | P42704 | 51 | 159003 | 3 | 3 | 3 | 3 | 0.09 | sp\|P42704\|LPPRC_HUMAN Leucine-rich PPR motif-containing protein, mitochondrial OS=Homo sapiens GN=LRPPRC PE=1 SV=3 |
| 130 | 1 | uniprot_human | Q86T13 | 21 | 52857 | 1 | 1 | 1 | 1 | 0.09 | sp\|Q86T13\|CLC14_HUMAN C-type lectin domain family 14 member A OS=Homo sapiens GN=CLEC14A PE=1 SV=1 |
| 63 | 1 | uniprot_human | Q8TB61 | 41 | 48054 | 1 | 1 | 1 | 1 | 0.09 | sp\|Q8TB61\|S35B2_HUMAN Adenosine 3'-phospho 5'-phosphosulfate transporter 1 OS=Homo sapiens GN=SLC35B2 PE=1 SV=1 |
| 98 | 1 | uniprot_human | A0A024RCG2 | 29 | 45244 | 1 | 1 | 1 | 1 | 0.1 | tr\|A0A024RCG2\|A0A024RCG2_HUMAN Solute carrier family 22 (Organic cation transporter), member 18, isoform CRA_a OS=Homo sapiens GN=SLC22A18 PE=1 SV=1 |
| 169 | 1 | uniprot_human | A0A0J9YWQ2 | 15 | 46497 | 1 | 1 | 1 | 1 | 0.1 | tr\|A0A0J9YWQ2\|A0A0J9YWQ2_HUMAN Phosphatidylserine synthase 2 (Fragment) OS=Homo sapiens GN=PTDSS2 PE=4 SV=1 |
| 114 | 1 | uniprot_human | H3BRG4 | 25 | 44778 | 1 | 1 | 1 | 1 | 0.1 | tr\|H3BRG4\|H3BRG4_HUMAN Cytochrome b-c1 complex subunit 2, mitochondrial OS=Homo sapiens GN=UQCRC2 PE=1 SV=1 |
| 158 | 1 | uniprot_human | J3QLD9 | 16 | 47569 | 1 | 1 | 1 | 1 | 0.1 | tr\|J3QLD9\|J3QLD9_HUMAN Flotillin-2 OS=Homo sapiens GN=FLOT2 PE=1 SV=1 |
| 99 | 1 | uniprot_human | P20020 | 28 | 139637 | 3 | 3 | 3 | 3 | 0.1 | sp\|P20020\|AT2B1_HUMAN Plasma membrane calcium-transporting ATPase 1 OS=Homo sapiens GN=ATP2B1 PE=1 SV=3 |
| 104 | 1 | uniprot_human | A0A087WU53 | 27 | 41961 | 1 | 1 | 1 | 1 | 0.11 | tr\|A0A087WU53\|A0A087WU53_HUMAN Magnesium transporter protein 1 OS=Homo sapiens GN=MAGT1 PE=1 SV=1 |
| 170 | 1 | uniprot_human | H3BR70 | 15 | 40506 | 1 | 1 | 1 | 1 | 0.11 | tr\|H3BR70\|H3BR70_HUMAN Pyruvate kinase OS=Homo sapiens GN=PKM PE=1 SV=1 |
| 117 | 1 | uniprot_human | H0Y6E9 | 24 | 38125 | 1 | 1 | 1 | 1 | 0.12 | tr\|H0Y6E9\|H0Y6E9_HUMAN Palmitoyltransferase (Fragment) OS=Homo sapiens GN=ZDHHC16 PE=3 SV=1 |
| 118 | 1 | uniprot_human | H3BM89 | 24 | 37861 | 1 | 1 | 1 | 1 | 0.12 | tr\|H3BM89\|H3BM89_HUMAN 60S ribosomal protein L4 OS=Homo sapiens GN=RPL4 PE=1 SV=1 |
| 101 | 1 | uniprot_human | I3NI36 | 28 | 39451 | 1 | 1 | 1 | 1 | 0.12 | tr\|I3NI36\|I3NI36_HUMAN A-kinase anchor protein 1, mitochondrial (Fragment) OS=Homo sapiens GN=AKAP1 PE=1 SV=1 |
| 18 | 1 | uniprot_human | P02751 | 147 | 266052 | 8 | 8 | 7 | 7 | 0.12 | sp\|P02751\|FINC_HUMAN Fibronectin OS=Homo sapiens GN=FN1 PE=1 SV=4 |
| 77 | 1 | uniprot_human | P19338 | 33 | 76625 | 2 | 2 | 2 | 2 | 0.12 | sp\|P19338\|NUCL_HUMAN Nucleolin OS=Homo sapiens GN=NCL PE=1 SV=3 |
| 138 | 1 | uniprot_human | Q9UJS0 | 20 | 74528 | 2 | 2 | 2 | 2 | 0.12 | sp\|Q9UJS0\|CMC2_HUMAN Calcium-binding mitochondrial carrier protein Aralar2 OS=Homo sapiens GN=SLC25A13 PE=1 SV=2 |
| 70 | 1 | uniprot_human | P05089 | 35 | 34884 | 1 | 1 | 1 | 1 | 0.13 | sp\|P05089\|ARGI1_HUMAN Arginase-1 OS=Homo sapiens GN=ARG1 PE=1 SV=2 |
| 157 | 1 | uniprot_human | P05198 | 16 | 36374 | 1 | 1 | 1 | 1 | 0.13 | sp\|P05198\|IF2A_HUMAN Eukaryotic translation initiation factor 2 subunit 1 OS=Homo sapiens GN=EIF2S1 PE=1 SV=3 |
| 6 | 3 | uniprot_human | P11021 | 127 | 72402 | 3 | 3 | 2 | 2 | 0.13 | sp\|P11021\|GRP78_HUMAN 78 kDa glucose-regulated protein OS=Homo sapiens GN=HSPA5 PE=1 SV=2 |
| 40 | 1 | uniprot_human | Q14739 | 68 | 71057 | 2 | 2 | 2 | 2 | 0.13 | sp\|Q14739\|LBR_HUMAN Lamin-B receptor OS=Homo sapiens GN=LBR PE=1 SV=2 |
| 171 | 1 | uniprot_human | Q8NGI4 | 15 | 35573 | 1 | 1 | 1 | 1 | 0.13 | sp\|Q8NGI4\|OR4DB_HUMAN Olfactory receptor 4D11 OS=Homo sapiens GN=OR4D11 PE=3 SV=1 |
| 75 | 1 | uniprot_human | A0A087WV81 | 34 | 32590 | 1 | 1 | 1 | 1 | 0.14 | tr\|A0A087WV81\|A0A087WV81_HUMAN Mannose-P-dolichol utilization defect 1 protein OS=Homo sapiens GN=MPDU1 PE=1 SV=1 |
| 79 | 1 | uniprot_human | C9IZQ1 | 33 | 33866 | 1 | 1 | 1 | 1 | 0.14 | tr\|C9IZQ1\|C9IZQ1_HUMAN Translocon-associated protein subunit alpha OS=Homo sapiens GN=SSR1 PE=1 SV=1 |
| 106 | 1 | uniprot_human | Q96HS1 | 27 | 32213 | 1 | 1 | 1 | 1 | 0.14 | sp\|Q96HS1\|PGAM5_HUMAN Serine/threonine-protein phosphatase PGAM5, mitochondrial OS=Homo sapiens GN=PGAM5 PE=1 SV=2 |
| 83 | 1 | uniprot_human | B5MCW2 | 32 | 31452 | 1 | 1 | 1 | 1 | 0.15 | tr\|B5MCW2\|B5MCW2_HUMAN 60S ribosomal protein L3 (Fragment) OS=Homo sapiens GN=RPL3 PE=1 SV=1 |
| 176 | 1 | uniprot_human | C9J826 | 14 | 30415 | 1 | 1 | 1 | 1 | 0.15 | tr\|C9J826\|C9J826_HUMAN Junction plakoglobin (Fragment) OS=Homo sapiens GN=JUP PE=1 SV=6 |
| 28 | 1 | uniprot_human | D6REL8 | 97 | 31616 | 2 | 2 | 1 | 1 | 0.15 | tr\|D6REL8\|D6REL8_HUMAN Fibrinogen beta chain OS=Homo sapiens GN=FGB PE=1 SV=1 |
| 72 | 1 | uniprot_human | F5H0G0 | 35 | 30427 | 1 | 1 | 1 | 1 | 0.15 | tr\|F5H0G0\|F5H0G0_HUMAN ATP-binding cassette sub-family B member 9 OS=Homo sapiens GN=ABCB9 PE=1 SV=1 |
| 52 | 1 | uniprot_human | H0YB39 | 49 | 30590 | 2 | 2 | 1 | 1 | 0.15 | tr\|H0YB39\|H0YB39_HUMAN Heterogeneous nuclear ribonucleoprotein H (Fragment) OS=Homo sapiens GN=HNRNPH1 PE=1 SV=1 |
| 96 | 1 | uniprot_human | H7C2G2 | 29 | 31719 | 1 | 1 | 1 | 1 | 0.15 | tr\|H7C2G2\|H7C2G2_HUMAN NAD(P)(+)--arginine ADP-ribosyltransferase (Fragment) OS=Homo sapiens GN=ART4 PE=1 SV=1 |
| 51 | 1 | uniprot_human | P10809 | 51 | 61187 | 2 | 2 | 2 | 2 | 0.15 | sp\|P10809\|CH60_HUMAN 60 kDa heat shock protein, mitochondrial OS=Homo sapiens GN=HSPD1 PE=1 SV=2 |
| 26 | 2 | uniprot_human | P14625 | 59 | 92696 | 3 | 3 | 3 | 3 | 0.15 | sp\|P14625\|ENPL_HUMAN Endoplasmin OS=Homo sapiens GN=HSP90B1 PE=1 SV=1 |
| 91 | 1 | uniprot_human | P53701 | 29 | 30981 | 1 | 1 | 1 | 1 | 0.15 | sp\|P53701\|CCHL_HUMAN Cytochrome c-type heme lyase OS=Homo sapiens GN=HCCS PE=1 SV=1 |
| 53 | 1 | uniprot_human | P62701 | 48 | 29807 | 1 | 1 | 1 | 1 | 0.15 | sp\|P62701\|RS4X_HUMAN 40S ribosomal protein S4, X isoform OS=Homo sapiens GN=RPS4X PE=1 SV=2 |
| 92 | 1 | uniprot_human | E9PIE4 | 29 | 29044 | 1 | 1 | 1 | 1 | 0.16 | tr\|E9PIE4\|E9PIE4_HUMAN Mitochondrial carrier homolog 2 (Fragment) OS=Homo sapiens GN=MTCH2 PE=1 SV=6 |
| 107 | 1 | uniprot_human | Q9BSF4 | 27 | 29500 | 1 | 1 | 1 | 1 | 0.16 | sp\|Q9BSF4\|CS052_HUMAN Uncharacterized protein C19orf52 OS=Homo sapiens GN=C19orf52 PE=1 SV=2 |
| 110 | 1 | uniprot_human | B3KY94 | 26 | 26670 | 1 | 1 | 1 | 1 | 0.17 | tr\|B3KY94\|B3KY94_HUMAN CDP-diacylglycerol--inositol 3-phosphatidyltransferase OS=Homo sapiens GN=CDIPT PE=1 SV=1 |
| 162 | 1 | uniprot_human | C9JRZ6 | 16 | 26963 | 1 | 1 | 1 | 1 | 0.17 | tr\|C9JRZ6\|C9JRZ6_HUMAN MICOS complex subunit MIC19 OS=Homo sapiens GN=CHCHD3 PE=1 SV=1 |
| 24 | 1 | uniprot_human | F8W7S5 | 103 | 84674 | 3 | 3 | 3 | 3 | 0.17 | tr\|F8W7S5\|F8W7S5_HUMAN Ribosome-binding protein 1 OS=Homo sapiens GN=RRBP1 PE=1 SV=1 |
| 45 | 1 | uniprot_human | P53985 | 56 | 54593 | 2 | 2 | 2 | 2 | 0.17 | sp\|P53985\|MOT1_HUMAN Monocarboxylate transporter 1 OS=Homo sapiens GN=SLC16A1 PE=1 SV=3 |
| 19 | 1 | uniprot_human | Q01650 | 133 | 55659 | 3 | 3 | 2 | 2 | 0.17 | sp\|Q01650\|LAT1_HUMAN Large neutral amino acids transporter small subunit 1 OS=Homo sapiens GN=SLC7A5 PE=1 SV=2 |
| 56 | 1 | uniprot_human | Q5RI18 | 46 | 27803 | 1 | 1 | 1 | 1 | 0.17 | tr\|Q5RI18\|Q5RI18_HUMAN Heterogeneous nuclear ribonucleoprotein U (Fragment) OS=Homo sapiens GN=HNRNPU PE=1 SV=6 |
| 65 | 1 | uniprot_human | B1APP6 | 38 | 26397 | 1 | 1 | 1 | 1 | 0.18 | tr\|B1APP6\|B1APP6_HUMAN ATP-dependent 6-phosphofructokinase, platelet type (Fragment) OS=Homo sapiens GN=PFKP PE=1 SV=1 |
| 25 | 1 | uniprot_human | C9JC84 | 103 | 52932 | 3 | 3 | 2 | 2 | 0.18 | tr\|C9JC84\|C9JC84_HUMAN Fibrinogen gamma chain OS=Homo sapiens GN=FGG PE=1 SV=1 |
| 69 | 1 | uniprot_human | E9PKU4 | 35 | 25806 | 1 | 1 | 1 | 1 | 0.18 | tr\|E9PKU4\|E9PKU4_HUMAN 60S ribosomal protein L8 (Fragment) OS=Homo sapiens GN=RPL8 PE=1 SV=1 |
| 141 | 1 | uniprot_human | P00403 | 19 | 25719 | 1 | 1 | 1 | 1 | 0.18 | sp\|P00403\|COX2_HUMAN Cytochrome c oxidase subunit 2 OS=Homo sapiens GN=MT-CO2 PE=1 SV=1 |
| 109 | 1 | uniprot_human | A8MUD9 | 26 | 24474 | 1 | 1 | 1 | 1 | 0.19 | tr\|A8MUD9\|A8MUD9_HUMAN 60S ribosomal protein L7 OS=Homo sapiens GN=RPL7 PE=1 SV=1 |
| 146 | 1 | uniprot_human | C9J6Q8 | 18 | 24481 | 1 | 1 | 1 | 1 | 0.19 | tr\|C9J6Q8\|C9J6Q8_HUMAN Transmembrane protein 98 (Fragment) OS=Homo sapiens GN=TMEM98 PE=1 SV=1 |
| 127 | 1 | uniprot_human | M0R1Y2 | 21 | 25113 | 1 | 1 | 1 | 1 | 0.19 | tr\|M0R1Y2\|M0R1Y2_HUMAN ER lumen protein-retaining receptor (Fragment) OS=Homo sapiens GN=KDELR1 PE=1 SV=1 |
| 41 | 1 | uniprot_human | A0A087WVQ9 | 62 | 48195 | 3 | 3 | 2 | 2 | 0.2 | tr\|A0A087WVQ9\|A0A087WVQ9_HUMAN Elongation factor 1-alpha 1 OS=Homo sapiens GN=EEF1A1 PE=1 SV=1 |
| 126 | 1 | uniprot_human | B4DP62 | 22 | 23423 | 1 | 1 | 1 | 1 | 0.2 | tr\|B4DP62\|B4DP62_HUMAN Solute carrier family 25 (Mitochondrial carrier citrate transporter), member 1, isoform CRA_b OS=Homo sapiens GN=SLC25A1 PE=1 SV=1 |
| 108 | 1 | uniprot_human | J3KTE4 | 26 | 23347 | 1 | 1 | 1 | 1 | 0.2 | tr\|J3KTE4\|J3KTE4_HUMAN Ribosomal protein L19 OS=Homo sapiens GN=RPL19 PE=1 SV=1 |
| 44 | 1 | uniprot_human | O15173 | 57 | 23861 | 1 | 1 | 1 | 1 | 0.2 | sp\|O15173\|PGRC2_HUMAN Membrane-associated progesterone receptor component 2 OS=Homo sapiens GN=PGRMC2 PE=1 SV=1 |
| 161 | 1 | uniprot_human | A0A087WWI8 | 16 | 22146 | 1 | 1 | 1 | 1 | 0.21 | tr\|A0A087WWI8\|A0A087WWI8_HUMAN Coiled-coil domain-containing protein 113 OS=Homo sapiens GN=CCDC113 PE=4 SV=1 |
| 39 | 1 | uniprot_human | H7C463 | 69 | 68305 | 3 | 3 | 3 | 3 | 0.21 | tr\|H7C463\|H7C463_HUMAN MICOS complex subunit MIC60 (Fragment) OS=Homo sapiens GN=IMMT PE=1 SV=1 |
| 8 | 1 | uniprot_human | P78527 | 359 | 473749 | 24 | 24 | 21 | 21 | 0.21 | sp\|P78527\|PRKDC_HUMAN DNA-dependent protein kinase catalytic subunit OS=Homo sapiens GN=PRKDC PE=1 SV=3 |
| 90 | 1 | uniprot_human | Q5T8U3 | 29 | 21702 | 1 | 1 | 1 | 1 | 0.22 | tr\|Q5T8U3\|Q5T8U3_HUMAN 60S ribosomal protein L7a (Fragment) OS=Homo sapiens GN=RPL7A PE=1 SV=1 |
| 133 | 1 | uniprot_human | Q8IZ57 | 21 | 21746 | 1 | 1 | 1 | 1 | 0.22 | sp\|Q8IZ57\|NRSN1_HUMAN Neurensin-1 OS=Homo sapiens GN=NRSN1 PE=1 SV=1 |
| 154 | 1 | uniprot_human | A0A087WUV8 | 17 | 20643 | 1 | 1 | 1 | 1 | 0.23 | tr\|A0A087WUV8\|A0A087WUV8_HUMAN Basigin OS=Homo sapiens GN=BSG PE=1 SV=1 |
| 88 | 1 | uniprot_human | A0A087X163 | 31 | 20692 | 1 | 1 | 1 | 1 | 0.23 | tr\|A0A087X163\|A0A087X163_HUMAN Ras-related protein Rab-18 OS=Homo sapiens GN=RAB18 PE=1 SV=1 |
| 147 | 1 | uniprot_human | H7C1V0 | 18 | 20587 | 1 | 1 | 1 | 1 | 0.23 | tr\|H7C1V0\|H7C1V0_HUMAN Cathepsin D (Fragment) OS=Homo sapiens GN=CTSD PE=1 SV=1 |
| 26 | 1 | uniprot_human | P08238 | 102 | 83554 | 4 | 4 | 4 | 4 | 0.23 | sp\|P08238\|HS90B_HUMAN Heat shock protein HSP 90-beta OS=Homo sapiens GN=HSP90AB1 PE=1 SV=4 |
| 54 | 1 | uniprot_human | H3BPZ1 | 48 | 40271 | 2 | 2 | 2 | 2 | 0.24 | tr\|H3BPZ1\|H3BPZ1_HUMAN Very-long-chain (3R)-3-hydroxyacyl-CoA dehydratase 3 OS=Homo sapiens GN=HACD3 PE=1 SV=1 |
| 46 | 1 | uniprot_human | Q3ZCQ8 | 55 | 39850 | 2 | 2 | 2 | 2 | 0.24 | sp\|Q3ZCQ8\|TIM50_HUMAN Mitochondrial import inner membrane translocase subunit TIM50 OS=Homo sapiens GN=TIMM50 PE=1 SV=2 |
| 22 | 1 | uniprot_human | H0Y4S6 | 106 | 19165 | 12 | 12 | 1 | 1 | 0.25 | tr\|H0Y4S6\|H0Y4S6_HUMAN Cohesin subunit SA-3 (Fragment) OS=Homo sapiens GN=STAG3 PE=1 SV=1 |
| 119 | 1 | uniprot_human | K7EQQ1 | 24 | 18732 | 1 | 1 | 1 | 1 | 0.25 | tr\|K7EQQ1\|K7EQQ1_HUMAN Cleft lip and palate transmembrane protein 1 (Fragment) OS=Homo sapiens GN=CLPTM1 PE=1 SV=1 |
| 55 | 2 | uniprot_human | O75477 | 22 | 39072 | 2 | 2 | 2 | 2 | 0.25 | sp\|O75477\|ERLN1_HUMAN Erlin-1 OS=Homo sapiens GN=ERLIN1 PE=1 SV=1 |
| 30 | 1 | uniprot_human | P21980 | 86 | 78420 | 4 | 4 | 4 | 4 | 0.25 | sp\|P21980\|TGM2_HUMAN Protein-glutamine gamma-glutamyltransferase 2 OS=Homo sapiens GN=TGM2 PE=1 SV=2 |
| 81 | 1 | uniprot_human | S4R468 | 33 | 19186 | 1 | 1 | 1 | 1 | 0.25 | tr\|S4R468\|S4R468_HUMAN Calcium uniporter protein, mitochondrial (Fragment) OS=Homo sapiens GN=MCU PE=1 SV=1 |
| 55 | 1 | uniprot_human | E5RHW4 | 48 | 37929 | 2 | 2 | 2 | 2 | 0.26 | tr\|E5RHW4\|E5RHW4_HUMAN Erlin-2 (Fragment) OS=Homo sapiens GN=ERLIN2 PE=1 SV=1 |
| 174 | 1 | uniprot_human | P47914 | 14 | 17798 | 1 | 1 | 1 | 1 | 0.27 | sp\|P47914\|RL29_HUMAN 60S ribosomal protein L29 OS=Homo sapiens GN=RPL29 PE=1 SV=2 |
| 134 | 1 | uniprot_human | J3QR48 | 21 | 16502 | 1 | 1 | 1 | 1 | 0.29 | tr\|J3QR48\|J3QR48_HUMAN Importin subunit beta-1 (Fragment) OS=Homo sapiens GN=KPNB1 PE=1 SV=6 |
| 132 | 1 | uniprot_human | A0A075B7A0 | 21 | 16245 | 1 | 1 | 1 | 1 | 0.3 | tr\|A0A075B7A0\|A0A075B7A0_HUMAN 60S ribosomal protein L18 OS=Homo sapiens GN=RPL18 PE=1 SV=1 |
| 167 | 1 | uniprot_human | K7EJY8 | 15 | 16000 | 1 | 1 | 1 | 1 | 0.3 | tr\|K7EJY8\|K7EJY8_HUMAN Galectin-3-binding protein (Fragment) OS=Homo sapiens GN=LGALS3BP PE=1 SV=1 |
| 21 | 1 | uniprot_human | P31327 | 121 | 165975 | 10 | 10 | 10 | 10 | 0.3 | sp\|P31327\|CPSM_HUMAN Carbamoyl-phosphate synthase [ammonia], mitochondrial OS=Homo sapiens GN=CPS1 PE=1 SV=2 |
| 15 | 1 | uniprot_human | Q92542 | 172 | 79103 | 7 | 7 | 5 | 5 | 0.32 | sp\|Q92542\|NICA_HUMAN Nicastrin OS=Homo sapiens GN=NCSTN PE=1 SV=2 |
| 120 | 1 | uniprot_human | A0A075B6F6 | 23 | 30314 | 2 | 2 | 2 | 2 | 0.33 | tr\|A0A075B6F6\|A0A075B6F6_HUMAN Minor histocompatibility antigen H13 (Fragment) OS=Homo sapiens GN=HM13 PE=1 SV=1 |
| 27 | 1 | uniprot_human | P25705 | 100 | 59828 | 4 | 4 | 4 | 4 | 0.34 | sp\|P25705\|ATPA_HUMAN ATP synthase subunit alpha, mitochondrial OS=Homo sapiens GN=ATP5A1 PE=1 SV=1 |
| 36 | 1 | uniprot_human | H3BNP9 | 74 | 14184 | 1 | 1 | 1 | 1 | 0.35 | tr\|H3BNP9\|H3BNP9_HUMAN Sulfide:quinone oxidoreductase, mitochondrial (Fragment) OS=Homo sapiens GN=SQRDL PE=1 SV=1 |
| 143 | 1 | uniprot_human | M0QZC5 | 19 | 14103 | 1 | 1 | 1 | 1 | 0.35 | tr\|M0QZC5\|M0QZC5_HUMAN 40S ribosomal protein S11 OS=Homo sapiens GN=RPS11 PE=1 SV=1 |
| 16 | 1 | uniprot_human | E7EUT5 | 159 | 28024 | 3 | 3 | 2 | 2 | 0.36 | tr\|E7EUT5\|E7EUT5_HUMAN Glyceraldehyde-3-phosphate dehydrogenase OS=Homo sapiens GN=GAPDH PE=1 SV=1 |
| 42 | 1 | uniprot_human | P62851 | 59 | 13791 | 1 | 1 | 1 | 1 | 0.36 | sp\|P62851\|RS25_HUMAN 40S ribosomal protein S25 OS=Homo sapiens GN=RPS25 PE=1 SV=1 |
| 32 | 1 | uniprot_human | P04843 | 77 | 68641 | 5 | 5 | 5 | 5 | 0.37 | sp\|P04843\|RPN1_HUMAN Dolichyl-diphosphooligosaccharide--protein glycosyltransferase subunit 1 OS=Homo sapiens GN=RPN1 PE=1 SV=1 |
| 43 | 1 | uniprot_human | A0A0B4J1V0 | 59 | 13089 | 2 | 2 | 1 | 1 | 0.38 | tr\|A0A0B4J1V0\|A0A0B4J1V0_HUMAN Protein IGHV3-15 (Fragment) OS=Homo sapiens GN=IGHV3-15 PE=1 SV=1 |
| 95 | 1 | uniprot_human | H0YB22 | 29 | 13220 | 1 | 1 | 1 | 1 | 0.38 | tr\|H0YB22\|H0YB22_HUMAN 40S ribosomal protein S14 (Fragment) OS=Homo sapiens GN=RPS14 PE=1 SV=1 |
| 100 | 1 | uniprot_human | M0R1M6 | 28 | 13023 | 1 | 1 | 1 | 1 | 0.38 | tr\|M0R1M6\|M0R1M6_HUMAN Ubiquitin-60S ribosomal protein L40 (Fragment) OS=Homo sapiens GN=UBA52 PE=1 SV=1 |
| 47 | 1 | uniprot_human | M0R1S5 | 51 | 13033 | 2 | 2 | 1 | 1 | 0.38 | tr\|M0R1S5\|M0R1S5_HUMAN Vesicular glutamate transporter 1 (Fragment) OS=Homo sapiens GN=SLC17A7 PE=1 SV=1 |
| 163 | 1 | uniprot_human | A0A075B6H7 | 15 | 12889 | 1 | 1 | 1 | 1 | 0.39 | tr\|A0A075B6H7\|A0A075B6H7_HUMAN Protein IGKV3-7 (Fragment) OS=Homo sapiens GN=IGKV3-7 PE=4 SV=1 |
| 94 | 1 | uniprot_human | A0A096LNV8 | 29 | 12754 | 1 | 1 | 1 | 1 | 0.39 | tr\|A0A096LNV8\|A0A096LNV8_HUMAN Transmembrane protein 262 OS=Homo sapiens GN=TMEM262 PE=4 SV=1 |
| 78 | 1 | uniprot_human | B1AKQ8 | 33 | 12304 | 1 | 1 | 1 | 1 | 0.41 | tr\|B1AKQ8\|B1AKQ8_HUMAN Guanine nucleotide-binding protein G(I)/G(S)/G(T) subunit beta-1 (Fragment) OS=Homo sapiens GN=GNB1 PE=1 SV=6 |
| 93 | 1 | uniprot_human | E5RI98 | 29 | 11915 | 1 | 1 | 1 | 1 | 0.42 | tr\|E5RI98\|E5RI98_HUMAN Nucleophosmin (Fragment) OS=Homo sapiens GN=NPM1 PE=1 SV=1 |
| 116 | 1 | uniprot_human | H0YFC6 | 25 | 11786 | 1 | 1 | 1 | 1 | 0.43 | tr\|H0YFC6\|H0YFC6_HUMAN GTP-binding nuclear protein Ran (Fragment) OS=Homo sapiens GN=RAN PE=1 SV=1 |
| 35 | 1 | uniprot_human | Q5JP53 | 75 | 48135 | 4 | 4 | 4 | 4 | 0.43 | tr\|Q5JP53\|Q5JP53_HUMAN Tubulin beta chain OS=Homo sapiens GN=TUBB PE=1 SV=1 |
| 136 | 1 | uniprot_human | E5RGR9 | 20 | 11303 | 2 | 2 | 1 | 1 | 0.45 | tr\|E5RGR9\|E5RGR9_HUMAN Protein YIPF5 (Fragment) OS=Homo sapiens GN=YIPF5 PE=1 SV=1 |
| 175 | 1 | uniprot_human | I3L3B4 | 14 | 11247 | 1 | 1 | 1 | 1 | 0.45 | tr\|I3L3B4\|I3L3B4_HUMAN Uncharacterized protein (Fragment) OS=Homo sapiens PE=4 SV=1 |
| 13 | 1 | uniprot_human | P16615 | 211 | 116336 | 11 | 11 | 10 | 10 | 0.45 | sp\|P16615\|AT2A2_HUMAN Sarcoplasmic/endoplasmic reticulum calcium ATPase 2 OS=Homo sapiens GN=ATP2A2 PE=1 SV=1 |
| 58 | 1 | uniprot_human | A0A087WYW6 | 44 | 22702 | 2 | 2 | 2 | 2 | 0.46 | tr\|A0A087WYW6\|A0A087WYW6_HUMAN Coiled-coil domain-containing protein 47 OS=Homo sapiens GN=CCDC47 PE=1 SV=1 |
| 105 | 1 | uniprot_human | M0R0F0 | 27 | 22548 | 2 | 2 | 2 | 2 | 0.46 | tr\|M0R0F0\|M0R0F0_HUMAN 40S ribosomal protein S5 (Fragment) OS=Homo sapiens GN=RPS5 PE=1 SV=1 |
| 82 | 1 | uniprot_human | A0A0A0MRQ5 | 32 | 10727 | 1 | 1 | 1 | 1 | 0.47 | tr\|A0A0A0MRQ5\|A0A0A0MRQ5_HUMAN Peroxiredoxin-1 OS=Homo sapiens GN=PRDX1 PE=1 SV=1 |
| 33 | 1 | uniprot_human | P36542 | 77 | 33032 | 3 | 3 | 3 | 3 | 0.48 | sp\|P36542\|ATPG_HUMAN ATP synthase subunit gamma, mitochondrial OS=Homo sapiens GN=ATP5C1 PE=1 SV=1 |
| 74 | 1 | uniprot_human | E9PJD9 | 34 | 10235 | 2 | 2 | 1 | 1 | 0.5 | tr\|E9PJD9\|E9PJD9_HUMAN 60S ribosomal protein L27a OS=Homo sapiens GN=RPL27A PE=1 SV=1 |
| 17 | 1 | uniprot_human | Q00325 | 157 | 40525 | 6 | 6 | 4 | 4 | 0.53 | sp\|Q00325\|MPCP_HUMAN Phosphate carrier protein, mitochondrial OS=Homo sapiens GN=SLC25A3 PE=1 SV=2 |
| 6 | 2 | uniprot_human | A0A0G2JIW1 | 268 | 70351 | 11 | 11 | 7 | 7 | 0.54 | tr\|A0A0G2JIW1\|A0A0G2JIW1_HUMAN Heat shock 70 kDa protein 1B OS=Homo sapiens GN=HSPA1B PE=1 SV=1 |
| 149 | 1 | uniprot_human | H0YNY9 | 18 | 9689 | 1 | 1 | 1 | 1 | 0.54 | tr\|H0YNY9\|H0YNY9_HUMAN Eukaryotic translation initiation factor 2-alpha kinase 4 (Fragment) OS=Homo sapiens GN=EIF2AK4 PE=1 SV=1 |
| 23 | 1 | uniprot_human | P49411 | 104 | 49852 | 6 | 6 | 5 | 5 | 0.54 | sp\|P49411\|EFTU_HUMAN Elongation factor Tu, mitochondrial OS=Homo sapiens GN=TUFM PE=1 SV=2 |
| 102 | 1 | uniprot_human | F2Z2C7 | 28 | 18088 | 2 | 2 | 2 | 2 | 0.6 | tr\|F2Z2C7\|F2Z2C7_HUMAN Protein transport protein Sec61 subunit alpha isoform 2 OS=Homo sapiens GN=SEC61A2 PE=1 SV=1 |
| 84 | 1 | uniprot_human | P62269 | 32 | 17708 | 2 | 2 | 2 | 2 | 0.61 | sp\|P62269\|RS18_HUMAN 40S ribosomal protein S18 OS=Homo sapiens GN=RPS18 PE=1 SV=3 |
| 129 | 1 | uniprot_human | D6RBL0 | 21 | 8106 | 1 | 1 | 1 | 1 | 0.66 | tr\|D6RBL0\|D6RBL0_HUMAN Transmembrane protein 165 OS=Homo sapiens GN=TMEM165 PE=1 SV=1 |
| 64 | 1 | uniprot_human | H0YCJ7 | 39 | 15108 | 2 | 2 | 2 | 2 | 0.75 | tr\|H0YCJ7\|H0YCJ7_HUMAN 40S ribosomal protein S3 (Fragment) OS=Homo sapiens GN=RPS3 PE=1 SV=1 |
| 131 | 1 | uniprot_human | H3BQR0 | 21 | 7319 | 1 | 1 | 1 | 1 | 0.75 | tr\|H3BQR0\|H3BQR0_HUMAN Snurportin-1 (Fragment) OS=Homo sapiens GN=SNUPN PE=1 SV=1 |
| 153 | 1 | uniprot_human | A0A0B4J1X5 | 17 | 13002 | 2 | 2 | 2 | 2 | 0.91 | tr\|A0A0B4J1X5\|A0A0B4J1X5_HUMAN Protein IGHV3-74 (Fragment) OS=Homo sapiens GN=IGHV3-74 PE=1 SV=1 |
| 10 | 2 | uniprot_human | P12236 | 196 | 33073 | 7 | 7 | 5 | 5 | 0.92 | sp\|P12236\|ADT3_HUMAN ADP/ATP translocase 3 OS=Homo sapiens GN=SLC25A6 PE=1 SV=4 |
| 140 | 1 | uniprot_human | D6RHF4 | 20 | 6022 | 1 | 1 | 1 | 1 | 0.96 | tr\|D6RHF4\|D6RHF4_HUMAN UDP-glucose 6-dehydrogenase (Fragment) OS=Homo sapiens GN=UGDH PE=1 SV=1 |
| 6 | 1 | uniprot_human | E9PKE3 | 360 | 68991 | 18 | 18 | 10 | 10 | 1 | tr\|E9PKE3\|E9PKE3_HUMAN Heat shock cognate 71 kDa protein OS=Homo sapiens GN=HSPA8 PE=1 SV=1 |
| 57 | 1 | uniprot_human | G3V1N2 | 44 | 11997 | 2 | 2 | 2 | 2 | 1.01 | tr\|G3V1N2\|G3V1N2_HUMAN HCG1745306, isoform CRA_a OS=Homo sapiens GN=HBA2 PE=1 SV=1 |
| 10 | 1 | uniprot_human | P05141 | 273 | 33059 | 9 | 9 | 6 | 6 | 1.18 | sp\|P05141\|ADT2_HUMAN ADP/ATP translocase 2 OS=Homo sapiens GN=SLC25A5 PE=1 SV=7 |
| 34 | 1 | uniprot_human | P16403 | 75 | 21352 | 5 | 5 | 4 | 4 | 1.23 | sp\|P16403\|H12_HUMAN Histone H1.2 OS=Homo sapiens GN=HIST1H1C PE=1 SV=2 |
